# Supplementary material for: Ethnic inequalities in mental and physical multimorbidity in women of reproductive age: a data linkage cohort study
Source: BMJ Open. 2022 Jul 14;12(7):e059257. doi: 10.1136/bmjopen-2021-059257 (PMC9295657; doi:10.1136/bmjopen-2021-059257)
Supplement: Supplementary data [file bmjopen-2021-059257supp001.pdf]

Table S1. Association between SMI and risk factors, diagnoses and health care contacts within the exposed cohort women in contact with mental health services.

	**SMI**<br>**Univariate logistical regression**<br>**OR, 95% CI**<br>**n=3,817**	**Multivariate logistic regression**<br>**Adjusted model for ethnicity & IMD**<br>**Adj OR, 95% CI**<br>**n=3, 399**
**Overweight**	2.07 (1.72-2.50)***<br>n= 3,285	1.80(1.48- 2.19) ***<br>n= 3,015
**Underweight**	0.93 (0.67- 1.31)<br>n= 3,285	0.86(0.60 - 1.22)<br>n= 3,015
**Smoking**	1.28 (1.08 - 1.51)**	1.41 (1.18 - 1.69)***
**Alcohol abuse**	0.88 (0.63 - 1.23)	0.95 (0.67 – 1.35)
**Drug use**	1.04 (0.72 - 1.50)	1.04 (0.71 - 1.52)
**Folate prescription**	2.72 (2.14 – 3.44) ***	2.20 (1.70 - 2.84) ***
**Vitamin D deficiency**	1.20 (0.93 – 1.55)	0.98 (0.75 – 1.30)
**LARC**	0.80 (0.58 - 1.09)	0.86 (0.62-1.20)
**Emergency contraception**	1.05 (0.77- 1.43)	1.02 (0.74 - 1.41)
**TOP**	0.55 (0.29 – 1.04)	0.55 (0.28 – 1.08)
Physical Health Diagnoses		
**Asthma**	0.96 (0.76 -1.23)	0.97 (0.75 - 1.26)
**Diabetes**	2.72 (1.92 – 3.86) ***	2.31 (1.58 – 3.36)***
**Hypertension**	2.28 (1.58- 3.29)***	1.71 (1.15 – 2.56) **
**Epilepsy**	1.06 (0.63 - 1.80)	1.04 (0.60 – 1.80)
**HIV**	0.62 (0.22- 1.78)	0.39 (0.13- 1.13)
**Hepatitis B/C**	1.14 (0.61- 2.16)	1.20 (0.63 – 2.31)
**PCOS**	0.69 (0.46- 1.05)	0.76 (0.49 - 1.16)
**Endometriosis**	0.55 (0.28- 1.11)	0.55 (0.27 – 1.13)
Multi-morbidity		
**Physical**	1.36 (0.9-1.9)	1.20 (0.88 – 1.82)
**Health care use**	**SMI**<br>**Univariate Negative binominal regression**<br>**IRR, 95% CI**<br>**n=3,817**	**Multivariate negative Binominal regression**<br>**Adjusted model for ethnicity& IMD**<br>**adj IRR, 95% CI**<br>**n=3, 399**
**Community MH F2F**	4.02 (3.53 – 4.57)***	4.05 (3.51 – 4.67) ***
**Inpatient MH days**	32.37 (19.50 - 53.84) ***	40.31 (21.70 – 74.88) ***
**GP consultations**	1.41 (1.30- 1.52)***	1.31 (1.21- 1.43) ***

*** $p<0.001$ ** $p<0.01$ * $p<0.05$
