# Supplementary material for: Ethnic inequalities in mental and physical multimorbidity in women of reproductive age: a data linkage cohort study
Source: BMJ Open. 2022 Jul 14;12(7):e059257. doi: 10.1136/bmjopen-2021-059257 (PMC9295657; doi:10.1136/bmjopen-2021-059257)
Supplement: Supplementary data [file bmjopen-2021-059257supp002.pdf]

Table S2. Association between ethnicity, risk factors, health diagnoses and clinical contacts.

Base (White British)	**Univariate logistical regressions** Odds Ratio (OR) 95% CI **(n=15,491)**			
	**White other**	**Black**	**Asian**	**Other**
**SMI**	0.98 (0.75 -1.27)	3.84(2.99-4.95)***	1.12(0.74-1.69)	1.58(1.22-2.05)**
**Depression**	0.53(0.48-0.60)***	0.84(0.73-0.97)*	0.56(0.46-0.69)***	1.01(0.90-1.13)
**Overweight**	1.12(1.00 - 1.25) * (n=12, 485)	5.00(4.37-5.73)*** (n=12, 485)	1.14 (0.95-1.37) (n=12, 485)	1.39(1.24-1.56)*** (n=12, 485)
**Underweight**	1.19 (0.98-1.45)* (n=12, 485)	1.85(1.41-2,43)*** (n=12, 485)	1.93(1.45-2.55)*** (n=12, 485)	1.25(1.00-1.54)* (n=12, 485)
**Smoking**	1.12 (1.02-1.23)*	0.64(0.56-0.73)***	0.49(0.41-0.59)***	1.17(1.05-1.30)**
**Alcohol abuse**	0.45(0.34-0.60)***	0.54(0.37-0.79)***	0.29(0.15-0.56)***	0.97 (0.74-1.27)
**Drug use**	0.55(0.38-0.79)***	0.97 (0.64-1.47)	0.32(0.14- 0.75)*	0.86(0.59-1.23)
**Vitamin D Deficiency**	0.90(0.71-1.15)	4.36(3.46-5.49)***	2.96(2.22-3.94)***	1.57(1.23-1.99)***
**LARC**	0.57(0.48-0.68)***	0.70(0.56-0.87)***	0.36(0.24-0.52)***	0.78(0.65-0.94)**
**Emergency contraception**	0.66(0.52-0.84)***	1.72(1.35-2.20)***	0.62(0.41-0.96) *	1.15(0.91-1.45)
**TOP**	0.59(0.40-0.87)**	1.51 (1.01-2.24)*	1.46(0.89-2.41)	1.10 (0.76-1.60)
**Antidepressant**	0.56(0.51-0.63)***	1.07(0.95-1.26)	0.56(0.47-0.67)***	1.02(0.92-1.34)
**Antipsychotic**	0.79(0.67-0.99)*	3.02(2.43-3.76)***	1.05 (0.76-1.44)	1.52(1.22-1.90)***
**Valproate**	0.70(0.42- 1.19)	3.23(2.01-5.20)***	0.87 (0.37-2.02)	1.58 (0.97- 2.55)
**Lithium**	0.71 (0.39-1.28)	2.16 (1.20- 3.86)**	0.80 (0.30-2.15)	1.20 (0.67 -2.55)
**Folate prescription**	0.99 (0.75-1.30)	4.36 (3.35- 5.67)***	1.83(1.26-2.65)**	1.60(1.21-2.11)**
**Asthma**	0.53(0.45-0.63)***	0.95(0.79-1.15)	0.71(0.55-0.93) *	1.16(0.99-1.35)
**Diabetes**	1.05(0.69-1.59)	5.27(3.59-7.34)***	3.01(1.85-4.88)***	1.45(0.95-2.22)
**Hypertension**	1.17(0.77-1.77)	9.80(6.77-14.17)***	1.91 (1.10-3.34)*	1.60(1.05-2.44)*
**Epilepsy**	0.42(0.25-0.75)**	1.19(0.70-2.03)***	0.80 (0.37-1.75)	1.39 (0.89-2.17)
**PCOS**	0.69(0.53-0.87)**	0.74(0.55-1.01)	0.79 (0.53-1.16)	0.96(0.75-1.22)
**Endometriosis**	0.86 (0.57 – 1.28)	1.76(1.14-2.71)*	1.21 (0.67 – 2.20)	1.54(1.04-2.28)*
**Multimorbidity**				
**Physical**	0.59(0.42-0.82) **	3.17(2.34-4.28)***	1.24(0.78-1.96)	1.06(0.76-1.47)
**Mental and Physical**	0.57(0.48-0.67)***	2.08(1.76-2.46)***	0.80(0.61-1.04)	1.34(1.14-1.57)***
**Health care contacts**	**Univariate Negative Binominal Regression IRR , 95% CI**			
**GP Consultations**	0.70(0.66-0.74)*** (n=15,491)	1.69(1.58-1.81)*** (n=15,491)	0.95(0.85-1.81) (n=15,491)	1.09(1.03- 1.16)** (n=15,491)
**CMHT f2f contacts**	0.68(0.58-0.80)*** (n=3,494)	1.52(1.28-1.81)*** (n=3,494)	0.80(0.61-1.04) (n=3,494)	1.06(0.91-1.24) (n=3,494)
**Inpatients days**	0.84 (0.43-1.62) (n=3,494)	4.50(2.21-9.19)*** (n=3,494)	1.13(0.37-3.77) (n=3,494)	1.97(1.04-3.09) * (n=3,494)
