# Supplementary material for: Ethnic inequalities in mental and physical multimorbidity in women of reproductive age: a data linkage cohort study
Source: BMJ Open. 2022 Jul 14;12(7):e059257. doi: 10.1136/bmjopen-2021-059257 (PMC9295657; doi:10.1136/bmjopen-2021-059257)
Supplement: Author's manuscript [file bmjopen-2021-059257.draft_revisions.pdf]

BMJ Open is committed to open peer review. As part of this commitment we make the peer review history of every article we publish publicly available.

When an article is published we post the peer reviewers' comments and the authors' responses online. We also post the versions of the paper that were used during peer review. These are the versions that the peer review comments apply to.

The versions of the paper that follow are the versions that were submitted during the peer review process. They are not the versions of record or the final published versions. They should not be cited or distributed as the published version of this manuscript.

BMJ Open is an open access journal and the full, final, typeset and author-corrected version of record of the manuscript is available on our site with no access controls, subscription charges or pay-per-view fees (http://bmjopen.bmj.com).

If you have any questions on BMJ Open's open peer review process please email info.bmjopen@bmj.com

BMJ Open

Ethnic inequalities in mental and physical multi-morbidity in women of reproductive age: a data linkage cohort study

Journal:	*BMJ Open*
Manuscript ID	bmjopen-2021-059257
Article Type:	Original research
Date Submitted by the Author:	13-Nov-2021
Complete List of Authors:	Catalao, Raquel; Institute of Psychiatry Psychology and Neuroscience Division of Academic Psychiatry<br>Dorrington, Sarah; Institute of Psychiatry Psychology and Neuroscience, Psychological Medicine<br>Pritchard, Megan; South London and Maudsley NHS Foundation Trust<br>Jewell, Amelia; South London and Maudsley NHS Foundation Trust<br>Broadbent, Matthew; South London and Maudsley NHS Foundation Trust<br>Ashworth, Mark; King's College London, Primary Care and Public Health Sciences<br>HATCH, STEPHANI; King's College London, Institute of Psychiatry, Psychological Medicine<br>Howard, Louise; King's College London, Institute of Psychiatry
Keywords:	MENTAL HEALTH, PUBLIC HEALTH, PRIMARY CARE

SCHOLARONE™
Manuscripts

I, the Submitting Author has the right to grant and does grant on behalf of all authors of the Work (as defined in the below author licence), an exclusive licence and/or a non-exclusive licence for contributions from authors who are: i) UK Crown employees; ii) where BMJ has agreed a CC-BY licence shall apply, and/or iii) in accordance with the terms applicable for US Federal Government officers or employees acting as part of their official duties; on a worldwide, perpetual, irrevocable, royalty-free basis to BMJ Publishing Group Ltd ("BMJ") its licensees and where the relevant Journal is co-owned by BMJ to the co-owners of the Journal, to publish the Work in this journal and any other BMJ products and to exploit all rights, as set out in our licence.

The Submitting Author accepts and understands that any supply made under these terms is made by BMJ to the Submitting Author unless you are acting as an employee on behalf of your employer or a postgraduate student of an affiliated institution which is paying any applicable article publishing charge ("APC") for Open Access articles. Where the Submitting Author wishes to make the Work available on an Open Access basis (and intends to pay the relevant APC), the terms of reuse of such Open Access shall be governed by a Creative Commons licence – details of these licences and which Creative Commons licence will apply to this Work are set out in our licence referred to above.

Other than as permitted in any relevant BMJ Author's Self Archiving Policies, I confirm this Work has not been accepted for publication elsewhere, is not being considered for publication elsewhere and does not duplicate material already published. I confirm all authors consent to publication of this Work and authorise the granting of this licence.

For peer review only

Ethnic inequalities in mental and physical multi-morbidity in women of reproductive age: a data linkage cohort study

Catalao R (0000-0003-3672-6359)**, Dorrington S, Pritchard M, Jewell A , Broadbent M, Ashworth M, Hatch SL, and Howard LM**

R Catalao, NIHR Academic Clinical Fellow, Section of Women's Mental Health, Institute of Psychiatry, Psychology and Neuroscience, King's College London and South London and Maudsley NHS Foundation Trust, 16 De Crespigny Park, London, SE5 8A

S Dorrington, Academic Clinical Lecturer, Department of Psychological Medicine at King's College London and South London and Maudsley NHS Foundation Trust, 16 De Crespigny Park, Camberwell, London, SE5 8A

M Pritchard, CRIS Training and Development Lead , NIHR Biomedical Research Centre, South London and Maudsley NHS Foundation Trust, 16 De Crespigny Park, London, SE5 8A

A Jewell, Clinical Data Linkage Service Lead, NIHR Biomedical Research Centre, South London and Maudsley NHS Foundation Trust, 16 De Crespigny Park, London, SE5 8A

Broadbent M, CRIS Clinical Informatics Lead at the NIHR Biomedical Research Centre, South London and Maudsley NHS Foundation Trust, 16 De Crespigny Park, London, SE5 8A

Ashworth M, Reader in Primary Care, Department of Primary Care, School of Population Health and Environmental Sciences, King's College London, Guy's Campus, Addison House, London, SE1 1UL

Hatch SL, Professor of Sociology and Epidemiology, Psychological Medicine Department, King's College London,, 16 De Crespigny Park, London, SE5 8A

Howard LM, Professor of Women's Mental Health at KCL, 16 De Crespigny Park, Camberwell, London SE5 8A
South London and Maudsley NHS Foundation Trust (SLaM), 16 De Crespigny Park, London, SE5 8A

Correspondence to: R Catalao, Raquel.catalao@kcl.ac.uk

Word count: 3,512

For peer review only

Objectives

Explore inequalities in risk factors, mental and physical health morbidity in non-pregnant women of reproductive age in contact with mental health services and how these vary per ethnicity.

Design

Retrospective cohort study

Setting

Data from Lambeth DataNet, anonymized primary care records of this ethnically diverse London borough, linked to anonymized electronic mental health records ("CRIS secondary care database").

Participants

Women aged 15-40 with an episode of secondary mental health care between January 2008 and December 2018 and no antenatal or postnatal read codes (n=3,817) and a 4:1 age-matched comparison cohort (n=14,532).

Main outcome measures

Preconception risk factors including low/high BMI, smoking, alcohol, substance misuse, micronutrient deficiencies and physical diagnoses.

Results

Women in contact with mental health services (whether with or without SMI diagnoses) had a higher prevalence of all risk factors and physical health diagnoses studied. Women from minority ethnic groups were less likely to be diagnosed with depression in primary care compared to White British women [adj OR 0.66 (0.55- 0.79) p<0.001] and Black women were more likely to have a severe mental illness [adj OR 2.79 (2.13- 3.64) p<0.001]. Black and Asian women were less likely to smoke or misuse substances and more likely to be vitamin D deficient. Black women were significantly more likely to be overweight [adj OR 3.47 (3.00- 4.01 p <0.001], be diagnosed with hypertension [adj OR 3.95 (2.67 – 5.85) p<0.00] and have two or more physical health conditions [adj OR 1.94 (1.41 – 2.68) p<0.001] than White British women.

Conclusions

Our results challenge the perspective that regular monitoring of physical health in primary care should be exclusively encouraged in people with a SMI diagnosis. The striking differences in multi-morbidity for women in contact with mental health services and those of ethnic minority groups emphasise a need of integrative models of care.

Strengths and limitations of this study

- Large sample of 18,349 women living in a ethnically diverse inner city borough

For peer review only

- Near complete coverage of primary care data in a London borough with good ethnicity recording allowed us to explore prevalence of multi-morbidity in non-pregnant women of reproductive age at the population level, a often neglected population group
- Linkage with mental health care services records enabled us to explore disparities in women in contact with mental health services
- Clinicians may be biased in recording risk factors and physical health diagnoses as there are lack of incentives to record in those without severe mental illness
- We excluded highly mobile populations and defined ethnicity in 5 broader groups potentially obscuring differences within the groups

Introduction

People with mental disorders have high rates of physical comorbidities, which contribute to premature mortality and ongoing health inequalities [1]. Similarly there is a well-established link between perinatal mental disorders and adverse pregnancy outcomes [2]. There are also striking inequalities in outcomes for Black women during the perinatal period [3], but few studies have focused on ethnic inequalities in physical and mental multi-morbidities in women, nor on the prevalence of the conditions that affect women's reproductive health. However, improving health earlier in women's lives could have benefits for women, their children and the health of future generations.

In this study we aimed to explore disparities in risk factors and physical health diagnoses in women of reproductive age in contact with mental health services compared to primary care controls and how these may vary by ethnicity.

We hypothesized that women in contact with mental health services, particularly those with severe mental illness (a diagnosis of schizophrenia, bipolar affective disorder and other psychoses) would have a higher prevalence of multi-morbidity in primary care after controlling for deprivation, and women from ethnic minority groups would be disproportionally affected.

Methods

Settings and Data Sources

Lambeth is an ethnically diverse borough, with a greater number of Black Caribbean and Black African residents, and fewer South Asian residents, than most other areas of London [4], and a larger proportion of younger adults compared to averages in London and England [5]. Lambeth has high levels of deprivation [6] and high turnover [5].

Anonymised primary care data were extracted on November 2020 from the computerised medical records of all general practices ($n = 48$) as part of Lambeth DataNet (LDN). This data set includes data from all registered patients including diagnoses and prescriptions, stored as Read codes (a standard vocabulary for clinicians to record patient findings and procedures, in health and social care IT systems across UK primary and secondary care) [7].

Data were extracted from LDN for the study period January 2008 to December 2018 (total number of registrations 855 742). Secondary care data came from the Clinical Record Interactive Search (CRIS) [8], an application allowing research access to pseudonymised electronic health record data

For peer review only

from the South London and the Maudsley NHS Foundation Trust (SLaM), Lambeth's mental health provider.

Data linkage

CRIS and LDN data were linked and stored by the SLaM Clinical Data Linkage Service, which provides a safe haven environment with strict governance arrangements. Data were linked using encrypted NHS numbers, which were subsequently removed and destroyed such that the linked dataset became fully anonymised.

Inclusion criteria for exposed cohort:

All women aged between 15 and 40 at study window start in contact with SLaM during the study window, with no antenatal or postnatal Read codes in their LDN record before window end, and registered in LDN for at least two years duration in the study window (2 years could be split over multiple registrations with gaps in between) with at least 1 year that occurred after the earliest ever SLaM start date irrespective of study window. Contact with SLaM was defined as an active episode of care including a face-to-face event.

Inclusion criteria for unexposed cohort:

4:1 exact age matched women from LDN with at least two year registration on LDN during study window; no antenatal nor postnatal related Read codes before window end and no SLaM mental health care receipt.

Measures

Age

Age at start of the study (i.e. 1st January 2008) was calculated from subtracting the year of birth from year of data extraction.

Ethnicity

Ethnicity was extracted from LDN self-reported ethnicity data fields. There were over 100 different codes for ethnicity and these were broadly categorized in five different groups (White British; White Other; Black; Asian and Mixed/Other), as defined in 2011 Census [4].

Index of Multiple Deprivation

Information on 768 Lower Super Output Areas (geographical areas of around 650 households) associated with earliest LDN registration in the window was used to determine level of social deprivation using the Index of Multiple Deprivation (IMD-2019) [9]. The IMD combines information from seven domains (income deprivation, employment, education skills and training, health deprivation and disability, crime, barriers to housing and services and living environment deprivation) to produce an overall relative measure of deprivation.

Mental Health Diagnoses

Data extracted from LDN included severe mental illness (SMI) status using Quality and Outcomes Framework (QOF) criteria which is an annual reward and incentive programme for all GP practices

For peer review only

in England [10]. Data extracted from CRIS included ICD-10 diagnosis consistent with the same SMI diagnoses: F20; F22; F23; F25; F28; F29 (schizophrenia and related disorders, schizoaffective disorders and delusional disorders); F30 ; F31 (mania and bipolar affective disorders); F32.3, F33.3 (psychotic depression). Thus a subgroup of the exposed cohort was defined as SMI if they were part of the SMI register on LDN or had an ICD-10 diagnosis of SMI recorded on CRIS.

Data extracted from LDN also included those on the Depression register, which is defined as patients who have an unresolved record of depression in their clinical record [10].

Physical Health Variables derived from Read codes

The following Read Codes were extracted if present during study window:

Body Mass Index (BMI): Women were defined as overweight during the study window if they had BMI value recorded of over 25.0 or had Read codes in register associated with BMI above 25 or obesity. Women were defined as underweight when they had a BMI <18.5 or Read codes in their register consistent with an anorexia nervosa diagnosis. When several BMI values were available we used the most recent.

Smoking: Read codes for current smoker

High risk alcohol use: AUDIT-C questionnaire score >=5 or Read codes for hazardous, harmful alcohol use or alcohol dependence

Drug use: Read codes for substance misuse

Folic acid prescription: Extracted via Dictionary of medicines and devices (DMD) codes [11] for all folic acid formulary preparations

Vitamin D deficiency: Read codes for vitamin D deficiency

Emergency and Long acting reversible contraception (EC and LARC): DMD and relevant Read codes for LARC and EC prescriptions.

Physical health diagnoses: Read codes for diagnoses including asthma, hypertension, diabetes, epilepsy, HIV, hepatitis B and C, endometriosis and Polycystic Ovarian Syndrome (PCOS).

Psychiatric drugs: DMD codes on LDN and prescriptions from CRIS for Valproate, Lithium, antidepressant and antipsychotic medication.

Health care use: Number of LDN recorded primary care consultations included consultations with a doctor or nurse as face-to-face GP appointments, emergency consultations, home visits, out of hours or walk in clinics. Number of CRIS recorded psychiatric inpatient days and number of face-to-face attended community contacts.

Mortality: Number of deaths was ascertained by calculating number of people with a date of death extracted from LDN at the time of data extraction.

Data Analysis

For peer review only

Data were analysed in Stata 15.0 for Mac after cleaning. Descriptive statistics were used to look for differences in demographic factors between exposed cohort and unexposed cohort.

Univariate and multivariate logistic and negative binominal regression analyses (adjusting for *a priori* determined confounders of IMD and ethnicity) were used to examine the rate of risk factors, diagnoses and health services contacts across groups (controls vs women in contact with mental health services) and a subgroup analysis within the exposed group (SMI vs non SMI diagnoses). A complete case analysis was also conducted to explore disparities in the outcomes above between different ethnic groups in primary care with adjustment for IMD, SMI diagnoses and age.

Patient and public involvement

The authors had no direct contact information of the study participants because anonymised clinical data were used in accordance with strict confidentiality guidelines. All patients have the choice to opt-out of their anonymised data being used. CRIS and LDN were developed with extensive service user involvement and adheres to strict governance frameworks managed by service users. No patient was involved in developing the hypothesis, research questions, plans for the study's design or writing of the results. Results will be disseminated to local public health structures including Lambeth Health Watch.

Results:

There were 3,817 women from LDN who were in contact with mental health services (Diagram 1). These women were more likely to be from Black ethnic groups [OR 1.48 (1.31-1.67) $p<0.01$] and live in areas of higher deprivation [OR 1.01 (1.01- 1.02) $p<0.001$] compared to the rest of the LDN cohort.

Diagram 1. Exposed cohort selection from Lambeth DataNet.

For peer review only

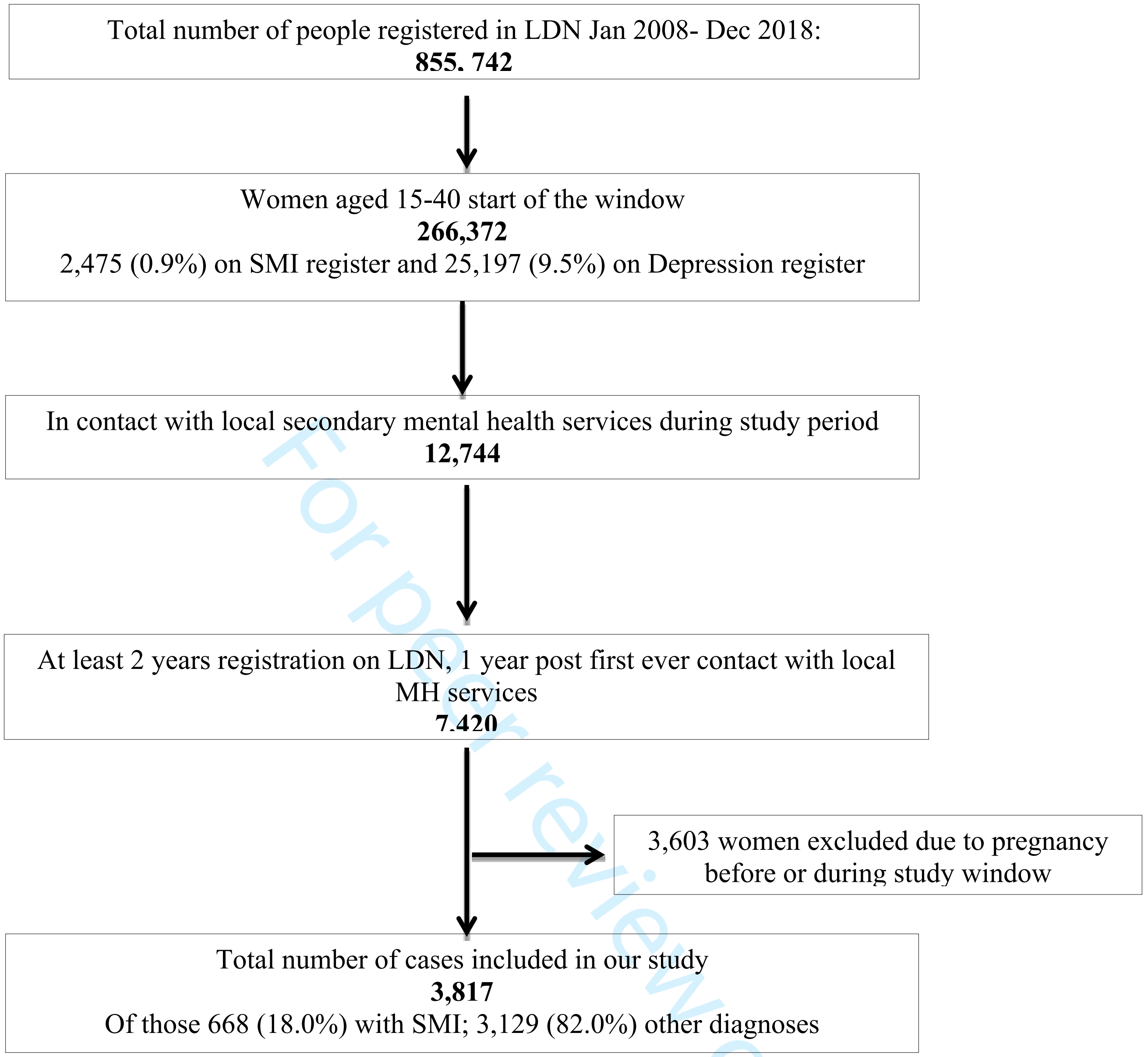

For peer review only

688 (18.0%) of the women in contact with mental care had a recorded SMI diagnosis; 420 (71.7%) of these women had recorded diagnoses of SMI on both LDN and CRIS; 102 (14.8%) women had a SMI diagnosis recorded on CRIS only and 166 women (24.1%) had SMI diagnosis recorded on LDN only. Subgroup analysis showed that women with SMI were more likely to be older and live in areas of higher deprivation (table 1). Women with SMI diagnosis recorded on LDN but not on CRIS had higher odds of substance misuse but not multi-morbidity.

Table 1. Characteristics of women of reproductive age in our sample: by contact with mental health services.

	**Primary care Controls** (n=14,532)		**In contact with local MH services** (n=3,817)		
		Missing	no SMI (n=3,129)	SMI diagnoses (n=688)	Missing

**Age**		0 (0.0%)			0 (0.0%)
15-19	3,030 (20.9%)		762 (25.7%)	111 (16.1%)	
20-24	3,430 (23.6%)		718 (24.2%)	128 (18.6%)	
25-29	2,706 (18.6%)		541 (18.2%)	115 (16.7%)	
30-34	2, 312 (15.9%)		442 (14.9%)	130 (18.9%)	
35-40	3, 054 (21.0%)		506(17.0%)	204 (29.6%)	
**Ethnicity**		2,535(17.4%)			323 (8.5%)
White British	2,467 (20.6%)		698 (24.5%)	92 (14.3%)	
Other white	4,615 (38.5%)		736 (25.8%)	152 (23.6%)	
Black	1,392 (11.6%)		452 (15.9%)	206 (32.0%))	
Asian	830 (6.9%)		149 (5.2%)	32 (5.0%)	
Other	2, 693 (22.5%)		816 (28.6%)	161 (25.0%)	
**IMD score**		621 (4.3%)			104 (2.7%)
1st quintile	2,211 (15.9%)		561 (18.4%)	153 (23.0%)	
2nd quintile	6,615 (47.6%)		1,490 (48.9%)	325 (48.9%)	
3rd quintile	3,783 (27.2%)		791 (26.0%)	158 (23.8%)	
4th quintile	1,078 (7.8%)		168 (5.5%)	27 (4.1%)	
5th (least deprived)	224 (1.6%)		38 (1.3%)	2 (0.3%)	
**Inpatient during study period (X^2)**	-		184 (5.9%)	305 (44.3%) ***	
**Number of inpatient days**					
Median test	-		Median 0 IQR[0-0]	Median 0 IQR [0 -61] ***	
**Number of days with attended CMHT f2f contacts**					
Median test	-		Median 5 IQR [1 – 21]	Median 37 IQR[7-111]***	
**Number of primary care clinical contacts**					
Median test	Median 13 IQR[2-34]		Median 59 IQR[27-110]	Median 89 IQR[37 – 157] ***	
**Mortality**	18 (0.1%)		45 (1.4%)	15 (2.2%)	

*** p<0.001 **p <0.01 *p<0.05

Missing data

Around 17.4% of women in the control group and 323 (8.5%) women in contact with mental health services had missing data on ethnicity. 621 (4.3%) of women in the control group and 104 women (2.7%) in women in contact with mental health (MH) services had missing data on IMD (table 1).

Missing data on ethnicity was associated with not being in contact with MH services, older age, living in areas of less deprivation and being a non-smoker; missing data on IMD was associated with younger age and Asian ethnicity.

532 (13.9%) women in contact with MH services and 4,582 (31.5%) in the comparison group had missing data on BMI; this was associated with not being in contact with MH services, older age and being of an ethnicity other than White British.

Inequalities in health outcomes

For peer review only

Women in contact with MH services vs primary care contact only

Women in contact with mental health services had a higher prevalence of all risk factors investigated (table 2 and 3). They were more likely to have a weight in the abnormal range - BMI>25 [adj OR 1.46 (1.33-1.60) p<0.0001] or BMI< 18.5 [adj OR 2.39 (2.05-2.19) p<0.001] (table 3). They had a higher prevalence of alcohol [adj OR 10.39 (8.10- 13.33) p<0.001] and substance misuse [adj OR 39.37 (23.87-64.86) p<0.001] after adjustment for ethnicity and IMD. They had higher rates of vitamin D deficiency, emergency contraception use and termination of pregnancy.

Table 2. Prevalence of risk factors and physical health disorders by contact with MH services and SMI status.

	Controls (n=14,532)	In contact with MH services (n= 3,817)	
Total = 18,349		**No SMI** (n=3,129)	**SMI diagnoses** (n=688)
**Overweight (BMI> 25) N=13,235**	3,086 (31.0%)	963 (36.3%)	343 (54.4%)
**Underweight (BMI < 18.5) N=13,235**	556 (5.6%)	299 (11.3%)	48 (7.6%)
**Smoker**	3,010 (20.7%)	1,322 (42.3%)	333 (48.4%)
**Excessive alcohol use**	92 (0.6%)	230 (7.4%)	45 (6.4%)
**Drug use**	19 (0.1%)	166 (5.3%)	38 (5.5%)
**Vitamin D deficiency**	469 (3.2%)	324 (10.4%)	84 (12.2%)
**Vitamin D prescription**	632 (4.4%)	525 (16.8%)	126 (18.3%)
**Folate prescription**	294 (2.0%)	230 (7.4%)	122 (17.7%)
**LARC Use**	655 (4.5%)	280 (8.9%)	50 (7.3%)
**Emergency Contraception**	374 (2.6%)	239 (7.6%)	55 (8.0%)
**TOP**	153 (1.0%)	89 (2.8%)	11 (1.6%)
**Psychiatric medication prescribed**			
**Valproate**	12 (0.1%)	29 (0.9%)	129 (18.8%)
**Antidepressants**	1,467 (10.1%)	2,045 (65.4%)	470 (68.3%)
**Antipsychotics**	51 (0.4%)	302 (9.7%)	536 (77.9%)
**Physical Health diagnoses**			
**Asthma**	851 (5.9%)	427 (13.7%)	91 (13.2%)
**Diabetes**	174 (1.2%)	93 (3.0%)	53 (7.7%)
**Hypertension**	258 (1.8%)	91 (3.0%)	45 (6.5%)
**Cardiovascular disease**	27 (0.2%)	7 (0.2%)	5 (0.7%)
**Epilepsy**	50 (0.3%)	77 (2.5%)	18 (2.6%)
**Endometriosis**	156 (1.1%)	73 (2.3%)	9 (1.3%)
**PCOS**	370 (2.6%)	174 (5.6%)	27 (3.9%)
**HIV**	19 (0.1%)	29 (0.9%)	4 (0.6%)

For peer review only

Hepatitis B/C	26 (0.2%)	48 (1.5%)	12 (1.7%)
**Multi-morbidity (2 or more long term conditions)**			
**Physical**	206 (1.4%)	269 (8.6%)	74 (10.8%)
**Mental and Physical**	395 (2.7%)	660 (21.1%)	428 (62.2%)-

Women in contact with mental health services had a higher prevalence of physical multi-morbidity (two or more physical health disorders) [9.0% vs 1.4%; adj OR 2.93 (2.37 – 3.63) p<0.001]. They had a particularly high prevalence of epilepsy [adj OR 5.73 (4.00- 8.20) p <0.001], HIV [adj OR 4.77 (2.66- 8.56) p<0.001] and chronic hepatitis [adj OR 8.24 (5.12- 13.24) p<0.001] (table 2 and 3).

Table 3. Association between contact with local MH services and risk factors, diagnoses and clinical contacts.

	**In contact with MH services Univariate logistical regression OR, 95% CI** (n=18,349)	**Multivariate logistic regression Adjusted model for Ethnicity and IMD Adj OR , 95% CI** (n=14,980)
**Overweight**	1.64 (1.50-1.78) *** n=13,235	1.46 (1.33 -1.60)*** n= 12,116
**Underweight**	2.41 (2.09-2.79)*** n=13,235	2.46(2.09-2.89)*** n=12,116
**Smoking**	2.93 (2.72-3.16)***	2.78(2.56-3.02)***
**Alcohol abuse**	12.19 (9.60-15.47) ***	10.39 (8.10- 13.33)***
**Drug use**	43.13 (26.91- 69.11)***	39.35 (23.87-64.86)***
**Folate prescription**	4.92 (4.19- 5.77)***	4.05 (3.42-4.80)***
**Vitamin D deficiency**	3.58 (3.12-4.11)***	2.92 (2.52- 3.39)***
**Vitamin D prescription**	4.52 (4.03- 5.08)***	3.71 (3.27-4.21)***
**LARC**	2.01 (1.75- 2.30) ***	1.67 (1.44- 1.94)***
**Emergency contraception**	3.16 (2.70- 3.70) ***	2.70 (2.28 -3.19)***
**TOP**	2.52 (1.96- 3.26) ***	2.10 (1.60- 2.76)***
Physical health diagnoses		**Adjusted model for Ethnicity and IMD Adj OR , 95% CI** (n=11,989)
**Asthma**	2.52 (2.25- 2.83)***	2.08 (1.84- 2.35)***
**Diabetes**	3.28 (2.62- 4.10) ***	2.55 (2.01- 3.24) ***
**Hypertension**	2.07 (1.68- 2.56) ***	1.39 (1.11 - 1.74) *
**Epilepsy**	7.39 (5.24- 10.43) ***	5.73 (4.00 - 8.20)***
**PCOS**	2.13 (1.78 -2.53) ***	1.75 (1.31 - 2.32)***
**Endometriosis**	2.02 (1.54- 2.65) ***	1.87 (1.55- 2.25)***
**HIV**	6.66 (3.78- 11.73)***	4.77 (2.66 – 8.56)**
**Hepatitis B/C**	8.91 (5.62 -14.14)***	8.24 (5.12 – 13.24)***
Mortality	12.88 (7.60- 21.83)***	16.10 (8.37- 30.91)***
Multi-morbidity		
**Physical**	4.09 (3.34- 5.00)***	2.93 (2.37 – 3.63)***

For peer review only

Mental and Physical	14.3 (12.6 – 16.1)***	11.7 (10.3 – 13.3)***
Health contacts	**Univariate negative binominal regression IRR, 95% CI** (n=18, 165)	**Adjusted model for SMI, age, IMD, ethnicity and Diabetes Adj IRR, 95%CI** (n=14,841)
**GP consultations**	3.47 (3.31- 3.64)***	2.92 (2.80-3.05)***

*** p<0.001 **p <0.01 *p<0.05

**Total (n= 15, 491)**	**White British (n= 3,357, 21.1%)**	**White other (n=5,503, 35.5%)**	**Black (n= 2,050, 13.2 %)**	**Asian (n=1,011, 6.5%)**	**Other (n=3,670, 23.7%)**
**SMI**	92 (2.8%)	152 (2.8%)	206 (10.1%)	32 (3.2%)	161 (4.4%)
**Depression**	660 (20.3%)	659 (12.0%)	361 (17.6%)	126 (12.5%)	748 (20.4%)
**Asthma**	320 (9.8%)	303 (5.5%)	193 (9.4%)	73 (7.2%)	411 (11.2%)
**Diabetes**	35 (1.1%)	62 (1.1%)	111 (5.4%)	32 (3.2%)	57 (1.6%)
**Hypertension**	34 (1.0%)	67 (1.2%)	192 (9.4%)	20 (2.0%)	61 (1.7%)
**Epilepsy**	32 (1.0%)	23 (0.4%)	24 (1.8%)	8 (0.8%)	50 (1.4%)
**Endometriosis**	40 (1.2%)	58 (1.1%)	44 (2.2%)	15 (1.5%)	69 (1.9%)

Only 28.4% of all patients prescribed valproate had a folic acid prescription issued in primary care. Similarly whereas women on valproate were more likely to be given LARC in primary care [OR 2.27 (1.40-3.68) p<0.001], only 10.6% of women on valproate were on LARC.

Women in contact with mental health services had a higher number of face-to-face clinical consultations in primary care during study period [adj IRR 2.74 (2.62-2.87) p<0.001] after adjusting for socio-demographic factors, SMI and a diabetes diagnosis. They were approximately sixteen times more likely to die [adj OR 16.10 (8.37 – 30.91) p<0.001] after adjusting for IMD and ethnicity and most deaths were among women without a SMI diagnosis (table 1 and table 3).

Subgroup analysis: SMI vs non SMI diagnoses in woman in contact with mental health services

Women with SMI were more likely to be overweight [adj OR 1.80 (1.48 -2.19) p<0.001] ; smokers [adj OR 1.41 (1.18- 1.69) p<0.001]; have a diagnosis of diabetes [adj OR 2.31(1.58- 3.36) p<0.001] and hypertension [adj OR 1.71 (1.15- 2.56) p<0.001] than other women in contact with mental health services with no SMI diagnoses after adjusting for IMD and ethnicity (supplementary file 1). They were also more likely to have a higher number of attended community mental health face to face appointments, inpatient days in mental health services, as well as GP face to face consultations during the study period.

Racial and Ethnic disparities

Mental Health

Across cases and controls with data on ethnicity (n=15, 491), there were significant discrepancies in diagnosis of depression in primary care. Fifteen percent of the whole sample (n=2,688) was on the primary care depression register. Women from White Other [adj OR 0.49 (0.44-0.56) p<0.001], Asian [adj OR 0.53 (0.43-0.65) p<0.001] and Black groups [adj OR 0.67 (0.57-0.78) p <0.001] were less likely to be diagnosed with depression in primary care compared to White British women after adjustment for age and IMD. Conversely, Black women were more likely to have a SMI diagnosis [adj OR 2.79 (2.13- 3.64) p<0.001] (table 4 and 5).

**PCOS**	131 (4.0%)	154 (2.8%)	62 (3.0%)	32 (3.2%)	142 (3.9%)
**HIV**	2 (0.1%)	8 (0.2%)	35 (1.7%)	0 (0.0%)	6 (0.2%)
**Hepatitis B/C**	17 (0.5%)	27 (0.5%)	15 (0.7%)	6 (0.6%)	21 (0.6%)
**Overweight (BMI>25) n=12,353**	730 (26.0%)	1,183(28.0%)	1,012 (62.2%)	206 (27.1%)	989 (32.3%)
**Underweight BMI<18.5 N=12,353**	172 (6.1%)	295 (7.0%)	88 (5.4%)	82 (10.8%)	209 (6.8%)
**Smoker**	921 (28.3%)	1,685 (30.6%)	414 (20.2%)	165 (16.3%)	1,157(31.5%)
**Alcohol abuse**	107 (3.3%)	83 (1.5%)	33 (1.7%)	9 (1.1%)	114(2.8%)
**Drug use**	59 (1.8%)	58 (1.1%)	44 (2.2%)	15 (1.5%)	69 (1.9%)
**Vit D deficiency**	109 (3.4%)	167 (3.0%)	269 (13.1%)	94 (9.3%)	189 (5.2%)
**LARC use**	265 (8.1%)	264 (4.8%)	119 (5.8%)	31 (3.1%)	238 (6.5%)
**EHC**	132 (4.1%)	150 (2.7%)	139 (6.8%)	26 (2.6%)	170 (4.6%)
**TOP**	51 (1.6%)	51 (0.9%)	48 (2.3%)	23 (2.3%)	63 (1.7%)
**Antipsychotic use**	135 (4.1%)	181 (3.3%)	237 (11.6%)	44 (4.4%)	227 (6.2%)
**Antidepressant use**	903 (27.7%)	981 (17.8%)	596 (29.1%)	178 (17.6%)	1,035 (28.2%)
**Physical multi-morbidity**	68 (2.1%)	68 (1.2%)	130 (6.3%)	26 (2.6%)	81 (2.2%)
**Physical and mental multi-morbidity**	286 (8.8%)	286 (5.2%)	342 (16.7%)	72 (7.1%)	419 (11.4%)
**Number of contacts in primary care**	Median 26 IQR[12-54]	Median 14 IQR [4-38]	Median 43 IQR [12-101]	Median 18 IQR[5 -53]	Median 28 IQR [11-60]
**Number of contacts in CMHT**	Median 9 IQR[1- 30]	Median 4 IQR[1- 25]	Median 10 IQR[1- 43]	Median 5 IQR[1 – 24]	Median 7 IQR[1- 32]
**Mortality**	21 (0.6%)	15 (0.3%)	20 (1.0%)	5 (0.5%)	8 (0.2%)

Table 4. Prevalence of mental and physical health diagnoses, risk factors and clinical contacts by ethnicity in total sample.

For peer review only

Table 5. Association between ethnicity, risk factors, health diagnoses and clinical contacts.

Base (White British)	**Univariate logistical regressions** Odds Ratio (OR) 95% CI **(n=15,491)**				**Multivariate logistical regressions** Adj Odds Ratio (OR) ; 95% CI **Model adjusted for IMD and age (ordinal) (n=14, 980)**			
	**White other**	**Black**	**Asian**	**Other**	**White other**	**Black**	**Asian**	**Other**
**SMI**	0.98 (0.75 -1.27)	3.84(2.99-4.95)***	1.12(0.74-1.69)	1.58(1.22-2.05)**	0.88 (0.64 – 1.09)	2.79 (2.13- 3.64)***	1.07 (0.71-1.61)	1.42 (1.09-1.85)**
**Depression**	0.53(0.48-0.60)***	0.84(0.73-0.97)*	0.56(0.46-0.69)***	1.01(0.90-1.13)	0.49 (0.44-0.56)***	0.67 (0.57-0.78)***	0.53(0.43-0.65)***	0.95 (0.84 – 1.07)
					**Model adjusted for IMD, SMI and age (n=14, 980)**			
**Overweight**	1.12(1.00 - 1.25) * (n=12, 485)	5.00(4.37-5.73)*** (n=12, 485)	1.14 (0.95-1.37) (n=12, 485)	1.39(1.24-1.56)*** (n=12, 485)	0.97 (0.86- 1.08) (n= 12, 116)	3.47 (3.00-4.01)*** (n= 12, 116)	0.96 (0.79 – 1.17) (n= 12,116)	1.24(1.10-1.40)*** (n= 12, 116)
**Underweight**	1.19 (0.98-1.45)* (n=12, 485)	1.85(1.41-2,43)*** (n=12, 485)	1.93(1.45-2.55)*** (n=12, 485)	1.25(1.00-1.54)* (n=12, 485)	1.22 (0.99 - 1.49) (n= 11,989)	1.79 (1.35-2.39)*** (n= 12, 116)	1.96(1.44 -2.63)*** (n= 12, 116)	1.28(1.03-1.59)* (n= 12, 116)
**Smoking**	1.12 (1.02-1.23)*	0.64(0.56-0.73)***	0.49(0.41-0.59)***	1.17(1.05-1.30)**	1.10(0.99 - 1.21)	0.53(0.46-0.61)***	0.47 (0.39-0.57)***	1.12(1.01-1.24)*
**Alcohol abuse**	0.45(0.34-0.60)***	0.54(0.37-0.79)***	0.29(0.15-0.56)***	0.97 (0.74-1.27)	0.38 (0.28 - 0.51)***	0.32 (0.21-0.49)***	0.26 (0.14-0.51)***	0.84 (0.64-1.10)
**Drug use**	0.55(0.38-0.79)***	0.97 (0.64-1.47)	0.32(0.14- 0.75)*	0.86(0.59-1.23)	0.46 (0.32-0.69)***	0.50 (0.32-0.79)**	0.28 (0.12-0.66)**	0.71 (0.49-1.03)
**Vitamin D Deficiency**	0.90(0.71-1.15)	4.36(3.46-5.49)***	2.96(2.22-3.94)***	1.57(1.23-1.99)***	0.79 (0.62-1.02)	3.03 (2.28- 3.87)***	2.72 (2.04 – 3.64)***	1.40 (1.09-1.78)***
**LARC**	0.57(0.48-0.68)***	0.70(0.56-0.87)***	0.36(0.24-0.52)***	0.78(0.65-0.94)**	0.56 (0.46-0.67)***	0.70 (0.56-0.89)**	0.36 (0.24-0.54)***	0.77(0.64-0.92)**
**Emergency contraception**	0.66(0.52-0.84)***	1.72(1.35-2.20)***	0.62(0.41-0.96) *	1.15(0.91-1.45)	0.72 (0.56-0.92)**	1.86 (1.43-2.42)***	0.64 (0.41-1.00)	1.19 (0.94-1.52)
**TOP**	0.59(0.40-0.87)**	1.51 (1.01-2.24)*	1.46(0.89-2.41)	1.10 (0.76-1.60)	0.60 (0.40- 0.90)*	1.68 (1.10 -2.56)*	1.57 (0.94 -2.65)	1.14 (0.78-1.66)
**Antidepressant**	0.56(0.51-0.63)***	1.07(0.95-1.26)	0.56(0.47-0.67)***	1.02(0.92-1.34)	0.52 (0.46-0.58)***	0.75 (0.65-0.86)***	0.51(0.42 -0.62)***	0.94 (0.84-1.05)
**Antipsychotic**	0.79(0.67-0.99)*	3.02(2.43-3.76)***	1.05 (0.76-1.44)	1.52(1.22-1.90)***	0.66 (0.49 –0.89)**	1.48 (1.08-2.04)*	0.91(0.57-1.45)	1.32 (0.99-1.76)
**Valproate**	0.70(0.42- 1.19)	3.23(2.01-5.20)***	0.87 (0.37-2.02)	1.58 (0.97- 2.55)	0.71 (0.41-1.24)	1.29 (0.76-2.26)	0.69 (0.27 – 1.75)	1.25 (0.74- 2.11)
**Lithium**	0.71 (0.39-1.28)	2.16 (1.20- 3.86)**	0.80 (0.30-2.15)	1.20 (0.67 -2.55)	0.68 (0.36-1.28)	0.65 (0.35-1.23)	0.69 (0.24- 1.96)	0.78 (0.41-1.45)
					**Model adjusted for IMD, SMI, valproate use and age (n=14, 841)**			
**Folate prescription**	0.99 (0.75-1.30)	4.36 (3.35- 5.67)***	1.83(1.26-2.65)***	1.60(1.21-2.11)***	0.86(0.64-1.14)	2.45 (1.84-3.25)***	1.67 (1.14-2.43)***	1.37 (1.03-1.82) *
					**Model adjusted for IMD, SMI and age**			

For peer review only - http://bmjopen.bmj.com/site/about/guidelines.xhtml

					(n= 11,989)			
**Asthma**	0.53(0.45-0.63)***	0.95(0.79-1.15)	0.71(0.55-0.93) *	1.16(0.99-1.35)	0.53(0.45-0.63)***	0.89 (0.73 – 1.09)	0.75 (0.57-0.98) *	1.14(0.97-1.33)
**Diabetes**	1.05(0.69-1.59)	5.27(3.59-7.34)***	3.01(1.85-4.88)***	1.45(0.95-2.22)	0.76 (0.50-1.16)	2.26 (1.51 -3.40)***	2.20 (1.34-3.60)**	1.05(0.68- 1.62))
**Hypertension**	1.17(0.77-1.77)	9.80(6.77-14.17)***	1.91 (1.10-3.34)*	1.60(1.05-2.44)*	0.83 (0.54- 1.27)	3.95 (2.67 – 5.85)***	1.33 (0.75- 2.35)	1.15 (0.74 – 1.77)
**Epilepsy**	0.42(0.25-0.75)**	1.19(0.70-2.03)***	0.80 (0.37-1.75)	1.39 (0.89-2.17)	0.36(0.21-0.62)***	0.63(0.38-1.20)	0.69 (0.32- 1.52)	1.17 (0.74- 1.84)
**PCOS**	0.69(0.53-0.87)**	0.74(0.55-1.01)	0.79 (0.53-1.16)	0.96(0.75-1.22)	0.80 (0.63- 1.02)	0.96 (0.69- 1.32)	0.97 (0.65 -1.44)	1.05 (0.82-1.34)
**Endometriosis**	0.86 (0.57 – 1.28)	1.76(1.14-2.71)*	1.21 (0.67 – 2.20)	1.54(1.04-2.28)*	0.80 (0.53-1.20)	1.46 (0.92 -2.32)	1.00 (0.73-2.32)	1.43 (0.96-2.13)
**Multimorbidit y**					**Model adjusted for IMD and age (n= 14, 980)**			
**Physical**	0.59(0.42-0.82) **	3.17(2.34-4.28)***	1.24(0.78-1.96)	1.06(0.76-1.47)	0.49 (0.34-0.69)***	1.94 (1.41-2.68)***	1.05 (0.66 – 1.68)	0.89 (0.64-1.24)
**Mental and Physical**	0.57(0.48-0.67)***	2.08(1.76-2.46)***	0.80(0.61-1.04)	1.34(1.14-1.57)***	0.98 (0.41-0.58)***	1.40 (1.17-1.67)***	0.71 (0.54 -0.94)*	1.19 (1.01-1.40) *
**Health care contacts**	**Univariate Negative Binominal Regression IRR , 95% CI**				**Multivariate Negative Binominal Regression Model adjusted for IMD, SMI and age adj IRR , 95% CI**			
**GP Consultations**	0.70(0.66-0.74)*** (n=15,491)	1.69(1.58-1.81)*** (n=15,491)	0.95(0.85-1.81) (n=15,491)	1.09(1.03- 1.16)** (n=15,491)	0.63 (0.60-0.66)*** (n=14,980)	1.26 (1.18-1.35)*** (n=14,980)	0.85 (0.78-0.93)*** (n=14,980)	1.09(0.95 -1.06) (n=14,980)
**CMHT f2f contacts**	0.68(0.58-0.80)*** (n=3,494)	1.52(1.28-1.81)*** (n=3,494)	0.80(0.61-1.04) (n=3,494)	1.06(0.91-1.24) (n=3,494)	0.59 (0.50-0.68)*** (n=3,399)	0.80 (0.68-0.95)* (n=3,399)	0.58(0.44 - 0.75)*** (n=3,399)	0.81(0.70-0.94)** (n=3,399)
**Inpatients days**	0.84 (0.43-1.62) (n=3,494)	4.50(2.21-9.19)*** (n=3,494)	1.13(0.37-3.77) (n=3,494)	1.97(1.04-3.09) * (n=3,494)	0.88 (0.46 -1. 67) (n=3,494)	0.75 (0.36 -1.56) (n=3,494)	0.92 (0.33- 2.53) (n=3,494)	1.75 (0.97 -3.15) (n=3,494)

For peer review only

Risk factors

Black women [adj OR 3.47 (3.00 – 4.01) p <0.001] and women from Mixed/Other ethnicities [adj OR 1.24 (1.10 -1.40) p<0.001] were significantly more likely to be overweight after adjusting for SMI status, IMD and age and Black, Asian and women from Mixed/ Other ethnicities were also more likely to have a BMI below 18.5 compared to White British women (table 5).

Both Black and Asian women were less likely to smoke, use alcohol excessively or misuse substances than White British women.

Black [adj OR 3.03(2.38- 3.87) p<0.001], Asian [adj OR 2.72 (2.04 – 3.64) p<0.001] and women from mixed and other ethnicities [adj OR 1.40 (1.09 -1.78) p<0.001] were more likely to be Vitamin D deficient. They were also more likely to be prescribed folic acid after adjustment for SMI, valproate use and age (table 5).

LARC prescription was low in our sample with less than 10% of women obtaining this method in primary care. Women from all ethnic minority groups were less likely to be prescribed or administered LARC in primary care compared to White British women, uptake was particularly low in Asian women [adj OR 0.36 (0.24-0.54) p<0.001]. Women from Black ethnic groups were also more likely to visit GP for emergency contraception [adj OR 1.64(1.27-2.12) p<0.001] and termination of pregnancy [adj OR 1.68 (1.10-2.56) p=0.016].

Psychiatric Medication

Black, Asian and women from White Other ethnic groups were less likely to be prescribed antidepressant medication in primary care compared to White British women. Whereas Black women were more likely to be prescribed antipsychotic medication after adjustment for SMI diagnoses, IMD and age.

Physical health diagnoses

Asthma was the most prevalent (7.5% of total sample) physical health diagnosis in all ethnic groups except in Black women where hypertension was the most prevalent diagnosis (9.4% of all Black women) (table 4).

Black women were almost four times more likely to be diagnosed with hypertension than White British women [adj OR 3.95 (2.67 – 5.85) p<0.001] .Diabetes was also more prevalent among Black [adj OR 2.26 (1.51- 3.40) p<0.001] and Asian women [adj OR 2.20 (1.34-3.60) p<0.001] compared to White British women (table 5).

The prevalence of PCOS and endometriosis was 3.1% and 1.3% of total sample respectively and there were no differences among different ethnic groups.

The numbers of women with HIV and hepatitis B/C in our sample were too small to conduct multivariate logistical regression analyses but the prevalence of HIV was disproportionally higher in Black women (1.7%) compared to whole sample prevalence (0.3%) (table 4).

Overall, Black women had higher rates of physical multi-morbidity compared to White British women [prevalence 6.3% vs 2.1%; adj OR 1.94 (1.41 –2.68) p<0.001] (table 4 and 5).

For peer review only

Health care contacts

Black women had a higher number of face-to-face primary care appointments during the study period than women from other ethnic groups after adjustment for IMD, SMI diagnoses and age [adj IRR 1.26 (1.18-1.35) p<0.001] (table 5).

After adjustment for SMI and IMD results showed that all ethnic minority groups had less community mental health face-to-face contacts compared to White British women.

Of the 15,491 women with data on ethnicity there were 69 (0.5%) deaths. The numbers were too small to perform logistical regression per ethnic group but there were significant differences with Black women having the higher risk of death (n =20, 1.0% fisher exact test p<0.001] (table 4).

Discussion

Principal findings

In this study using a primary care- secondary mental health care clinical data linkage of an ethnically diverse and deprived inner London borough, we found striking differences in risk factors, multi-morbidity and health service contacts by ethnicity and mental health service use for women of reproductive age. Women in contact with mental health services had higher prevalence of all physical health diagnoses and risk factors studied including a BMI outside the healthy range, smoking, alcohol, and substance misuse as well as micronutrient deficiencies. They also had higher rates of emergency contraception use and termination of pregnancy stressing a greater unmet need for contraception. Similarly to previous studies, we found the risk of HIV and chronic hepatitis [12] to be greatly elevated compared to controls. Adjusting for hypothesized exploratory factors including ethnicity, deprivation and SMI diagnosis only partly explained inequalities. The risk of all-cause mortality in this group was 16 times higher compared to controls and, notably, women without an SMI diagnosis were also at increased risk of death. Black women and women in contact with mental health services had a higher number of face-to-face clinical contacts in primary care so inequalities cannot be exclusively explained by lack of access.

Comparison with other studies

Our study adds to growing literature of inequalities in mental and physical health outcomes for women of ethnic minority groups in the UK. Our results echoes studies during the perinatal period which found women from ethnic minority groups are less likely to be diagnosed and receive treatment for depression in primary care [13] and paradoxically Black women are more likely to have an SMI diagnosis [14]. We found that Black and Asian women were less likely to engage in some risk behaviours, such as smoking or drinking alcohol excessively compared to White British but were more likely to have unhealthy weight. Black women were also more likely to be prescribed antipsychotics irrespective of diagnosis. There were also inequalities in sexual and reproductive health and women from ethnic minority groups had decreased uptake of LARC in primary care. Consistent with previous national survey data [15], Black women had higher use of emergency contraception than White British women. We also confirmed that Black women were at disproportionally high risk of hypertension compared to White British women [16].

Strengths and Weaknesses

For peer review only

To our knowledge this is one of the few studies investigating multi-morbidity in non-pregnant women of reproductive age at the population level and highlighting disparities by ethnicity and mental ill-health, with a large sample size and near complete coverage of primary care data in a London borough. Limitations include the exclusion of highly mobile populations not in contact with the same practice for 2 years, who may be at higher risk of poor health and under recording of risk factors and diagnoses (potentially due to clinicians' unconscious biases and lack of incentives to record risk factors in patients without SMI). Previous studies have found differences in multi-morbidity within the broader ethnic groups defined in this study (e.g. Black Caribbean and Black African) [17] but further disaggregation was not possible due to small cells. Our data on use of contraception must also be interpreted with caution, as these are patterns of use and access in primary care whereas there are alternative sources of these services in community sexual health clinics and pharmacies. National data has shown that women living in urban areas and from ethnic minority groups are more likely to visit sexual health clinics compared to White British women but primary care remains the main provider of contraception across population groups and inequalities persist despite the source of services [18]. Finally, we also did not have cause specific mortality and further research is required to elucidate this.

Policy implications

Our study highlights that striking differences in multimorbidity are present in ethnic minority groups with and without mental illness at the preconception stage which may partly explain inequalities in pregnancy outcomes and mortality for Black and Asian women during the perinatal period in the UK [19]. These differences suggest that there are major structural inequalities in healthcare provision in the UK. Our results also challenge the perspective that regular monitoring of physical health in primary care should be exclusively encouraged in people with a SMI diagnosis and indicate that there are mental and physical health needs of women in contact with mental health services that are not being met by the current model of service provision. Our study emphasizes the need for a more holistic approach to health promotion for women in contact with mental health services, expanding the remit beyond cardiovascular disease prevention into sexual and reproductive health, including avoidance of drugs with teratogenic potential and addressing micronutrient deficiencies and substance misuse.

Conclusions

There are striking inequalities in risk factor profile and health outcomes including mortality for women of reproductive age in contact with mental health services and those of ethnic minority groups emphasising a need of culturally centered integrative models of care. More attention should be focused on identifying missed opportunities to intervene across primary and secondary mental health services.

Summary boxes

What is already known about this topic

Women with mental health disorders and those of ethnic minority groups have worse pregnancy outcomes

There is lack of data measuring multi-morbidity in women of reproductive age at preconception stage and exploring ethnic disparities

For peer review only

What this study adds

Women of reproductive age in contact with mental health services whether with or without a severe mental illness diagnosis have a higher prevalence of physical multi-morbidity and unmet reproductive health needs

There are striking inequalities in the prevalence of mental and physical multi-morbidity in women of ethnic minority groups at preconception stage

Statements

Contributor and guarantor information:
RC conceived the research question and designed the study analysis plan with input from LMH and SLH. MP, AJ and MB performed data extraction. RC and SD analysed the data. RC wrote the first draft of the manuscript. LMH, SLH, MA and SD critically revised it. All authors approved the final version of the manuscript. The corresponding author attests that all listed authors meet authorship criteria and that no others meeting the criteria have been omitted.
The guarantor is RC, who affirms that the manuscript is an honest, accurate, and transparent account of the study being reported, that no important aspects of the study have been omitted, and that any discrepancies from the study as originally planned have been explained.

Acknowledgements

This work uses data provided by patients and collected by the NHS as part of their care and support . We are grateful for all the support received from the National Institute for Health Research (NIHR) BRC Maudsley clinical informatics teams throughout this work,

Financial support

This project has been funded by the Closing the Gap network. Closing the Gap is funded by UK Research and Innovation and their support is gratefully acknowledged (Grant reference: ES/S004459/1). The funding source had no role in project design, data analysis or writing the manuscript. This project was possible due to access to the data at the NIHR Maudsley Biomedical Research Centre at South London and Maudsley NHS Foundation Trust and King's College London. RC and SD receive salary support from NIHR as clinical academics. SLH receives support from the Economic and Social Research Council (ESRC), Medical Research Council (MRC), NIHR, Wellcome Trust and Guy's and St Thomas' Charity. LMH is a NIHR Principal Investigator and receives funding from NIHR Maudsley Biomedical Research Centre, NIHR Applied Research Collaboration South London and UKRI funded Network on Violence, Abuse and Mental Health . Any views expressed here are those of the project investigators and do not necessarily represent the views of the Closing the Gap network, UKRI, NIHR, ESRC, MRC, Wellcome Trust, NHS or King's College London.

Conflicts of Interest

All authors have completed a ICMJE disclosure form http://www.icmje.org/disclosure-of-interest/ and declare financial support from Closing the Gap Network and NIHR for the submitted work;

SLH receives consulting fees from the NHS Race and Health Observatory; no financial relationships with any organisations that might have an interest in the submitted work in the previous three years; All authors declare no other relationships or activities that could appear to have influenced the submitted work.

Ethical Standards and data sharing statement

Clinical Record Interactive Search (CRIS) was established in 2008 and approved by the Oxfordshire Research Ethics Committee in 2008 (reference 18/SC/0372). Approval for linkage with LDN was granted by Lambeth Clinical Commissioning Group and Information Governance Steering Group. The anonymised dataset that underlie the results in this article is only available on request to investigators whose proposed use of data has been approved by CRIS Oversight Committee and Lambeth Clinical Commissioning Group and Information Governance Steering Group. Complete list of codes used to identify study population and outcomes can be requested from the corresponding author (Raquel.catalao@kcl.ac.uk).

License for publication:
The Corresponding Author has the right to grant on behalf of all authors and does grant on behalf of all authors, a worldwide licence to the Publishers and its licensees in perpetuity, in all forms, formats and media (whether known now or created in the future), to i) publish, reproduce, distribute, display and store the Contribution, ii) translate the Contribution into other languages, create adaptations, reprints, include within colctions and create summaries, extracts and/or, abstracts of the Contribution, iii) create any other derivative work(s) based on the Contribution, iv) to exploit all subsidiary rights in the Contribution, v) the inclusion of electronic links from the Contribution to third party material where-ever it may be located; and, vi) licence any third party to do any or all of the above.

Provenance and peer review: Not commissioned; externally peer reviewed.

References:

1 Thornicroft G. Premature death among people with mental illness. BMJ. 2013;**346**. doi:10.1136/bmj.f2969

2 Stein A, Pearson RM, Goodman SH, *et al.* Effects of perinatal mental disorders on the fetus and child. *Lancet* 2014;**384**:1800–19. doi:10.1016/S0140-6736(14)61277-0

3 Knight M, Bunch K, Kenyon S, *et al.* A national population-based cohort study to investigate inequalities in maternal mortality in the United Kingdom, 2009-17. *Paediatr Perinat Epidemiol* 2020;**34**:392–8. doi:10.1111/ppe.12640

4 Office of National Statistics. 2011 Census: KS201 UK Ethnic group, local authorities in the United Kingdom. 2011. https://www.ons.gov.uk/peoplepopulationandcommunity/populationandmigration/populationestimates/datasets/2011censuskeystatisticsandquickstatisticsforlocalauthoritiesintheunitedkingdompart1 (accessed 1 Nov 2021)

5 Lambeth.gov.uk. Demography factsheet. 2017.https://www.lambeth.gov.uk/sites/default/files/ssh-demography-factsheet-2017.pdf (accessed 1 Nov 2021)

6 GOV.UK. English Indices of Deprivation 2010. 2010.https://www.gov.uk/government/statistics/english-indices-of-deprivation-2010 (accessed 1st November 2021)

For peer review only - http://bmjopen.bmj.com/site/about/guidelines.xhtml

7 NHS Digital. Read Codes - NHS Digital. https://digital.nhs.uk/services/terminology-and-classifications/read-codes (accessed 1 Nov 2021).
8 Stewart R, Soremekun M, Perera G, *et al.* The South London and Maudsley NHS Foundation Trust Biomedical Research Centre (SLAM BRC) case register: Development and descriptive data. *BMC Psychiatry* Published Online First: 2009. doi:10.1186/1471-244X-9-51
9 GOV.UK. English indices of deprivation 2019 - GOV.UK. https://www.gov.uk/government/statistics/english-indices-of-deprivation-2019 (accessed 1 Nov 2021).
10 NHS Digital. QOF 2019-20 | NHS Digital. https://qof.digital.nhs.uk/ (accessed 1 Nov 2021).
11 Dictionary of medicines and devices (dm+d) | NHSBSA. https://www.nhsbsa.nhs.uk/pharmacies-gp-practices-and-appliance-contractors/dictionary-medicines-and-devices-dmd (accessed 1 Nov 2021).
12 Hughes E, Bassi S, Gilbody S, *et al.* Prevalence of HIV, hepatitis B, and hepatitis C in people with severe mental illness: A systematic review and meta-analysis. *The Lancet Psychiatry* 2016;**3**:40–8. doi:10.1016/S2215-0366(15)00357-0
13 Prady SL, Pickett KE, Petherick ES, *et al.* Evaluation of ethnic disparities in detection of depression and anxiety in primary care during the maternal period: Combined analysis of routine and cohort data. *Br J Psychiatry* 2016;**208**:453–61. doi:10.1192/bjp.bp.114.158832
14 Abel KM, Hope H, Swift E, *et al.* Prevalence of maternal mental illness among children and adolescents in the UK between 2005 and 2017: a national retrospective cohort analysis. *Lancet Public Heal* 2019;**4**:e291–300. doi:10.1016/S2468-2667(19)30059-3
15 Wayal S, Hughes G, Sonnenberg P, *et al.* Ethnic variations in sexual behaviours and sexual health markers: findings from the third British National Survey of Sexual Attitudes and Lifestyles (Natsal-3). *Lancet Public Heal* 2017;**2**:e458–72. doi:10.1016/S2468-2667(17)30159-7
16 Primatesta P, Bost L, Poulter NR. Blood pressure levels and hypertension status among ethnic groups in England. *J Hum Hypertens* Published Online First: 2000. doi:10.1038/sj.jhh.1000960
17 Dorrington S, Carr E, Stevelink SAM, *et al.* Multimorbidity and fit note receipt in working-age adults with long-term health conditions. *Psychol Med* 2020;:1–10. doi:10.1017/S0033291720002937
18 French RS, Geary R, Jones K, *et al.* Where do women and men in Britain obtain contraception? Findings from the third National Survey of Sexual Attitudes and Lifestyles (Natsal-3). *BMJ Sex Reprod Heal* 2018;**44**:16–26. doi:10.1136/jfprhc-2017-101728
19 Knight M, Bunch K, Tuffnell D, *et al. Saving Lives, Improving Mothers' Care Maternal, Newborn and Infant Clinical Outcome Review Programme*. 2018.

For peer review only

Table S1. Association between SMI and risk factors, diagnoses and health care contacts within the exposed cohort women in contact with mental health services.

	**SMI**<br>**Univariate logistical regression**<br>**OR, 95% CI**<br>**n=3,817**	**Multivariate logistic regression**<br>**Adjusted model for ethnicity & IMD**<br>**Adj OR, 95% CI**<br>**n=3, 399**
**Overweight**	2.07 (1.72-2.50)***<br>n= 3,285	1.80(1.48- 2.19) ***<br>n= 3,015
**Underweight**	0.93 (0.67- 1.31)<br>n= 3,285	0.86(0.60 - 1.22)<br>n= 3,015
**Smoking**	1.28 (1.08 - 1.51)**	1.41 (1.18 - 1.69)***
**Alcohol abuse**	0.88 (0.63 - 1.23)	0.95 (0.67 – 1.35)
**Drug use**	1.04 (0.72 - 1.50)	1.04 (0.71 - 1.52)
**Folate prescription**	2.72 (2.14 – 3.44) ***	2.20 (1.70 - 2.84) ***
**Vitamin D deficiency**	1.20 (0.93 – 1.55)	0.98 (0.75 – 1.30)
**LARC**	0.80 (0.58 - 1.09)	0.86 (0.62-1.20)
**Emergency contraception**	1.05 (0.77- 1.43)	1.02 (0.74 - 1.41)
**TOP**	0.55 (0.29 – 1.04)	0.55 (0.28 – 1.08)
Physical Health Diagnoses		
**Asthma**	0.96 (0.76 -1.23)	0.97 (0.75 - 1.26)
**Diabetes**	2.72 (1.92 – 3.86) ***	2.31 (1.58 – 3.36)***
**Hypertension**	2.28 (1.58- 3.29)***	1.71 (1.15 – 2.56) **
**Epilepsy**	1.06 (0.63 - 1.80)	1.04 (0.60 – 1.80)
**HIV**	0.62 (0.22- 1.78)	0.39 (0.13- 1.13)
**Hepatitis B/C**	1.14 (0.61- 2.16)	1.20 (0.63 – 2.31)
**PCOS**	0.69 (0.46- 1.05)	0.76 (0.49 - 1.16)
**Endometriosis**	0.55 (0.28- 1.11)	0.55 (0.27 – 1.13)
Multi-morbidity		
**Physical**	1.36 (0.9-1.9)	1.20 (0.88 – 1.82)
**Health care use**	**SMI**<br>**Univariate Negative binominal regression**<br>**IRR, 95% CI**<br>**n=3,817**	**Multivariate negative Binominal regression**<br>**Adjusted model for ethnicity& IMD**<br>**adj IRR, 95% CI**<br>**n=3, 399**
**Community MH F2F**	4.02 (3.53 – 4.57)***	4.05 (3.51 – 4.67) ***
**Inpatient MH days**	32.37 (19.50 - 53.84) ***	40.31 (21.70 – 74.88) ***
**GP consultations**	1.41 (1.30- 1.52)***	1.31 (1.21- 1.43) ***

**** p<0.001 **p <0.01 *p<0.05*

For peer review only

The RECORD statement – checklist of items, extended from the STROBE statement, that should be reported in observational studies using routinely collected health data.

	Item No.	STROBE items	Location in manuscript where items are reported	RECORD items	Location in manuscript where items are reported
**Title and abstract**					
	1	(a) Indicate the study's design with a commonly used term in the title or the abstract (b) Provide in the abstract an informative and balanced summary of what was done and what was found	Page 1 and 2	RECORD 1.1: The type of data used should be specified in the title or abstract. When possible, the name of the databases used should be included.<br><br>RECORD 1.2: If applicable, the geographic region and timeframe within which the study took place should be reported in the title or abstract.<br><br>RECORD 1.3: If linkage between databases was conducted for the study, this should be clearly stated in the title or abstract.	Page 1 & 2<br><br>Page 1 & 2<br><br>Page 1 & 2
**Introduction**					
Background rationale	2	Explain the scientific background and rationale for the investigation being reported	Page 3		
Objectives	3	State specific objectives, including any prespecified hypotheses	Page 3		
**Methods**					
Study Design	4	Present key elements of study design early in the paper	page 1& 2		
Setting	5	Describe the setting, locations, and relevant dates, including periods of recruitment, exposure, follow-up, and data collection	Page 3		
Participants	6	*(a) Cohort study* - Give the eligibility criteria, and the	Page 4	RECORD 6.1: The methods of study population selection (such as codes or	Page 4 & page 18

For peer review only

		sources and methods of selection of participants. Describe methods of follow-up<br>*Case-control study* - Give the eligibility criteria, and the sources and methods of case ascertainment and control selection. Give the rationale for the choice of cases and controls<br>*Cross-sectional study* - Give the eligibility criteria, and the sources and methods of selection of participants<br><br>*(b) Cohort study* - For matched studies, give matching criteria and number of exposed and unexposed<br>*Case-control study* - For matched studies, give matching criteria and the number of controls per case	Page 4	algorithms used to identify subjects) should be listed in detail. If this is not possible, an explanation should be provided.<br><br>RECORD 6.2: Any validation studies of the codes or algorithms used to select the population should be referenced. If validation was conducted for this study and not published elsewhere, detailed methods and results should be provided.<br><br>RECORD 6.3: If the study involved linkage of databases, consider use of a flow diagram or other graphical display to demonstrate the data linkage process, including the number of individuals with linked data at each stage.	Diagram 1
Variables	7	Clearly define all outcomes, exposures, predictors, potential confounders, and effect modifiers. Give diagnostic criteria, if applicable.	Page 4 & 5 & 6	RECORD 7.1: A complete list of codes and algorithms used to classify exposures, outcomes, confounders, and effect modifiers should be provided. If these cannot be reported, an explanation should be provided.	Page 4 &5 & page 12
Data sources/ measurement	8	For each variable of interest, give sources of data and details of methods of assessment (measurement).<br>Describe comparability of assessment methods if there is more than one group	Page 4 & 5 & 6		
Bias	9	Describe any efforts to address potential sources of bias	Page 5 & 6		
Study size	10	Explain how the study size was	Page 6 ; Diagram 1		

		arrived at			
Quantitative variables	11	Explain how quantitative variables were handled in the analyses. If applicable, describe which groupings were chosen, and why	Page 5 & 6		
Statistical methods	12	(a) Describe all statistical methods, including those used to control for confounding<br>(b) Describe any methods used to examine subgroups and interactions<br>(c) Explain how missing data were addressed<br>(d) *Cohort study* - If applicable, explain how loss to follow-up was addressed<br>*Case-control study* - If applicable, explain how matching of cases and controls was addressed<br>*Cross-sectional study* - If applicable, describe analytical methods taking account of sampling strategy<br>(e) Describe any sensitivity analyses	Page 5 & 6		
Data access and cleaning methods		..		RECORD 12.1: Authors should describe the extent to which the investigators had access to the database population used to create the study population.<br><br>RECORD 12.2: Authors should provide information on the data cleaning methods used in the study.	Page 3 &4
Linkage		..		RECORD 12.3: State whether the study included person-level, institutional-	Page 4

For peer review only

				level, or other data linkage across two or more databases. The methods of linkage and methods of linkage quality evaluation should be provided.	
**Results**					
Participants	13	(a) Report the numbers of individuals at each stage of the study (*e.g.*, numbers potentially eligible, examined for eligibility, confirmed eligible, included in the study, completing follow-up, and analysed)<br>(b) Give reasons for non-participation at each stage.<br>(c) Consider use of a flow diagram	Page 6 & 7<br>Diagram 1	RECORD 13.1: Describe in detail the selection of the persons included in the study (*i.e.,* study population selection) including filtering based on data quality, data availability and linkage. The selection of included persons can be described in the text and/or by means of the study flow diagram.	Page 6 & 7<br>Diagram 1
Descriptive data	14	(a) Give characteristics of study participants (*e.g.*, demographic, clinical, social) and information on exposures and potential confounders<br>(b) Indicate the number of participants with missing data for each variable of interest<br>(c) *Cohort study* - summarise follow-up time (*e.g.*, average and total amount)	Page 6, 7 & 8<br><br>Table 1		
Outcome data	15	*Cohort study* - Report numbers of outcome events or summary measures over time<br>*Case-control study* - Report numbers in each exposure category, or summary measures of exposure<br>*Cross-sectional study* - Report numbers of outcome events or summary measures	Page 8-15		
Main results	16	(a) Give unadjusted estimates	Page 8- 15		

For peer review only

		and, if applicable, confounder-adjusted estimates and their precision (e.g., 95% confidence interval). Make clear which confounders were adjusted for and why they were included<br>(b) Report category boundaries when continuous variables were categorized<br>(c) If relevant, consider translating estimates of relative risk into absolute risk for a meaningful time period			
Other analyses	17	Report other analyses done—e.g., analyses of subgroups and interactions, and sensitivity analyses	Page 11		
**Discussion**					
Key results	18	Summarise key results with reference to study objectives	Page 16		
Limitations	19	Discuss limitations of the study, taking into account sources of potential bias or imprecision. Discuss both direction and magnitude of any potential bias	Page 16, 17	RECORD 19.1: Discuss the implications of using data that were not created or collected to answer the specific research question(s). Include discussion of misclassification bias, unmeasured confounding, missing data, and changing eligibility over time, as they pertain to the study being reported.	Page 16, 17
Interpretation	20	Give a cautious overall interpretation of results considering objectives, limitations, multiplicity of analyses, results from similar studies, and other relevant evidence	Page 17		
Generalisability	21	Discuss the generalisability (external validity) of the study results	page 17		

For peer review only

Other Information					
Funding	22	Give the source of funding and the role of the funders for the present study and, if applicable, for the original study on which the present article is based	Page 18		
Accessibility of protocol, raw data, and programming code		..		RECORD 22.1: Authors should provide information on how to access any supplemental information such as the study protocol, raw data, or programming code.	Page 18, 19

*Reference: Benchimol EI, Smeeth L, Guttmann A, Harron K, Moher D, Petersen I, Sørensen HT, von Elm E, Langan SM, the RECORD Working Committee. The REporting of studies Conducted using Observational Routinely-collected health Data (RECORD) Statement. *PLoS Medicine* 2015; in press.

*Checklist is protected under Creative Commons Attribution (CC BY) license.

For peer review only

BMJ Open

Ethnic inequalities in mental and physical multi-morbidity in women of reproductive age: a data linkage cohort study

Journal:	*BMJ Open*
Manuscript ID	bmjopen-2021-059257.R1
Article Type:	Original research
Date Submitted by the Author:	20-Apr-2022
Complete List of Authors:	Catalao, Raquel; King's College London Department of Psychological Medicine<br>Dorrington, Sarah; King's College London Department of Psychological Medicine, Psychological Medicine<br>Pritchard, Megan; South London and Maudsley NHS Foundation Trust<br>Jewell, Amelia; South London and Maudsley NHS Foundation Trust<br>Broadbent, Matthew; South London and Maudsley Mental Health NHS Trust<br>Ashworth, Mark; King's College London, Primary Care and Public Health Sciences<br>HATCH, STEPHANI; King's College London Department of Psychological Medicine<br>Howard, Louise; King's College London Institute of Psychiatry Psychology and Neuroscience
**Primary Subject Heading**:	Public health
Secondary Subject Heading:	Epidemiology, General practice / Family practice, Health informatics, Mental health
Keywords:	MENTAL HEALTH, PUBLIC HEALTH, PRIMARY CARE

SCHOLARONE™
Manuscripts

I, the Submitting Author has the right to grant and does grant on behalf of all authors of the Work (as defined in the below author licence), an exclusive licence and/or a non-exclusive licence for contributions from authors who are: i) UK Crown employees; ii) where BMJ has agreed a CC-BY licence shall apply, and/or iii) in accordance with the terms applicable for US Federal Government officers or employees acting as part of their official duties; on a worldwide, perpetual, irrevocable, royalty-free basis to BMJ Publishing Group Ltd ("BMJ") its licensees and where the relevant Journal is co-owned by BMJ to the co-owners of the Journal, to publish the Work in this journal and any other BMJ products and to exploit all rights, as set out in our licence.

The Submitting Author accepts and understands that any supply made under these terms is made by BMJ to the Submitting Author unless you are acting as an employee on behalf of your employer or a postgraduate student of an affiliated institution which is paying any applicable article publishing charge ("APC") for Open Access articles. Where the Submitting Author wishes to make the Work available on an Open Access basis (and intends to pay the relevant APC), the terms of reuse of such Open Access shall be governed by a Creative Commons licence – details of these licences and which Creative Commons licence will apply to this Work are set out in our licence referred to above.

Other than as permitted in any relevant BMJ Author's Self Archiving Policies, I confirm this Work has not been accepted for publication elsewhere, is not being considered for publication elsewhere and does not duplicate material already published. I confirm all authors consent to publication of this Work and authorise the granting of this licence.

For peer review only

Ethnic inequalities in mental and physical multi-morbidity in women of reproductive age: a data linkage cohort study

Catalao R (0000-0003-3672-6359)**, Dorrington S, Pritchard M, Jewell A , Broadbent M, Ashworth M, Hatch SL, and Howard LM**

R Catalao, NIHR Academic Clinical Fellow, Section of Women's Mental Health, Institute of Psychiatry, Psychology and Neuroscience, King's College London and South London and Maudsley NHS Foundation Trust, 16 De Crespigny Park, London, SE5 8A

S Dorrington, Academic Clinical Lecturer, Department of Psychological Medicine at King's College London and South London and Maudsley NHS Foundation Trust, 16 De Crespigny Park, Camberwell, London, SE5 8A

M Pritchard, CRIS Training and Development Lead , NIHR Biomedical Research Centre, South London and Maudsley NHS Foundation Trust, 16 De Crespigny Park, London, SE5 8A

A Jewell, Clinical Data Linkage Service Lead, NIHR Biomedical Research Centre, South London and Maudsley NHS Foundation Trust, 16 De Crespigny Park, London, SE5 8A

Broadbent M, CRIS Clinical Informatics Lead at the NIHR Biomedical Research Centre, South London and Maudsley NHS Foundation Trust, 16 De Crespigny Park, London, SE5 8A

Ashworth M, Reader in Primary Care, Department of Primary Care, School of Population Health and Environmental Sciences, King's College London, Guy's Campus, Addison House, London, SE1 1UL

Hatch SL, Professor of Sociology and Epidemiology, Department of Psychological Medicine and ESRC Centre for Society and Mental Health, King's College London, 16 De Crespigny Park, London, SE5 8A

Howard LM, Professor of Women's Mental Health at KCL, 16 De Crespigny Park, Camberwell, London SE5 8A
South London and Maudsley NHS Foundation Trust (SLaM), 16 De Crespigny Park, London, SE5 8A

Correspondence to: R Catalao, Raquel.catalao@kcl.ac.uk

Word count: 3,848

For peer review only

Objectives

Explore inequalities in risk factors, mental and physical health morbidity in non-pregnant women of reproductive age in contact with mental health services and how these vary per ethnicity.

Design

Retrospective cohort study

Setting

Data from Lambeth DataNet, anonymized primary care records of this ethnically diverse London borough, linked to anonymized electronic mental health records ("CRIS secondary care database").

Participants

Women aged 15-40 with an episode of secondary mental health care between January 2008 and December 2018 and no antenatal or postnatal read codes (n=3,817) and a 4:1 age-matched comparison cohort (n=14,532).

Main outcome measures

Preconception risk factors including low/high BMI, smoking, alcohol, substance misuse, micronutrient deficiencies and physical diagnoses.

Results

Women in contact with mental health services (whether with or without severe mental illness (SMI)) had a higher prevalence of all risk factors and physical health diagnoses studied. Women from minority ethnic groups were less likely to be diagnosed with depression in primary care compared to White British women [adj OR 0.66 (0.55- 0.79) p<0.001] and Black women were more likely to have a SMI [adj OR 2.79 (2.13- 3.64) p<0.001]. Black and Asian women were less likely to smoke or misuse substances and more likely to be vitamin D deficient. Black women were significantly more likely to be overweight [adj OR 3.47 (3.00- 4.01 p <0.001], be diagnosed with hypertension [adj OR 3.95 (2.67 – 5.85) p<0.00] and have two or more physical health conditions [adj OR 1.94 (1.41 – 2.68) p<0.001] than White British women.

Conclusions

Our results challenge the perspective that regular monitoring of physical health in primary care should be exclusively encouraged in people with a SMI diagnosis. The striking differences in multi-morbidity for women in contact with mental health services and those of ethnic minority groups emphasise a need of integrative models of care.

For peer review only

STRENGTHS AND LIMITATIONS OF THE STUDY

- Large sample of 18,349 women living in a ethnically diverse inner city borough
- Near complete coverage of primary care data in a London borough with good ethnicity recording allowed us to explore prevalence of multi-morbidity in non-pregnant women of reproductive age at the population level, a often neglected population group
- Linkage with mental health care services records enabled us to explore disparities in women in contact with mental health services
- Clinicians may be biased in recording risk factors and physical health diagnoses as there are lack of incentives to record in those without severe mental illness
- We excluded highly mobile populations and defined ethnicity in 5 broader groups potentially obscuring differences within the groups

INTRODUCTION

People with mental disorders have high rates of physical comorbidities, which contribute to premature mortality and ongoing health inequalities [1]. Similarly there is a well-established link between perinatal mental disorders and adverse pregnancy outcomes [2]. There are also striking inequalities in outcomes for Black women during the perinatal period [3], but few studies have focused on ethnic inequalities in physical and mental multi-morbidities in women, nor on the prevalence of the conditions that affect women's reproductive health. However, improving health earlier in women's lives could have benefits for women, their children and the health of future generations.

In this study we aimed to explore disparities in risk factors and physical health diagnoses in women of reproductive age in contact with mental health services compared to primary care controls and how these may vary by ethnicity.

We hypothesized that women in contact with mental health services, particularly those with severe mental illness (SMI; a diagnosis of schizophrenia, bipolar affective disorder and other psychoses) would have a higher prevalence of multi-morbidity in primary care after controlling for deprivation, and women from ethnic minority groups would be disproportionally affected.

METHODS

Settings and Data Sources

Lambeth is an ethnically diverse borough, with a greater number of Black Caribbean and Black African residents, and fewer South Asian residents, than most other areas of London [4], and a larger proportion of younger adults compared to averages in London and England [5]. Lambeth has high levels of deprivation [6] and high turnover [5].

Anonymised primary care data were extracted on November 2020 from the computerised medical records of all general practices ($n = 48$) as part of Lambeth DataNet (LDN). This data set includes data from all registered patients including diagnoses and prescriptions, stored as Read codes (a

For peer review only

standard vocabulary for clinicians to record patient findings and procedures, in health and social care IT systems across UK primary and secondary care) [7].

Data were extracted from LDN for the study period January 2008 to December 2018 (total number of registrations 855 742). Secondary care data came from the Clinical Record Interactive Search (CRIS) [8], an application allowing research access to pseudonymised electronic health record data from the South London and the Maudsley NHS Foundation Trust (SLaM), Lambeth's mental health provider.

Data linkage

CRIS and LDN data were linked and stored by the SLaM Clinical Data Linkage Service, which provides a safe haven environment with strict governance arrangements. Data were linked using encrypted NHS numbers, which were subsequently removed and destroyed such that the linked dataset became fully anonymised.

Inclusion criteria for exposed cohort:

Women aged between 15 and 40 at the start of the study window in contact with SLaM during the study window, defined as an active episode of care including a face-to-face event, were included in this study. Women were excluded from the exposed cohort if they had antenatal or postnatal Read Codes in their primary care record before window end and were registered in LDN for less than two years during in the study window. At least 1 year registration in LDN occurred after the earliest ever SLaM start date irrespective of study window.

Inclusion criteria for unexposed cohort:

4:1 exact age matched women from LDN with at least two year registration on LDN during study window; no antenatal nor postnatal related Read codes before window end and no SLaM mental health care receipt.

Measures

Age

Age at start of the study (i.e. 1st January 2008) was calculated from subtracting the year of birth from year of data extraction.

Ethnicity

Ethnicity was extracted from LDN self-reported ethnicity data fields. There were over 100 different codes for ethnicity and these were broadly categorized in five different groups (White British; White Other; Black; Asian and Mixed/Other), as defined in 2011 Census [4].

Index of Multiple Deprivation

Information on 768 Lower Super Output Areas (geographical areas of around 650 households) associated with earliest LDN registration in the window was used to determine level of social deprivation using the Index of Multiple Deprivation (IMD-2019) [9]. The IMD combines

For peer review only

information from seven domains (income deprivation, employment, education skills and training, health deprivation and disability, crime, barriers to housing and services and living environment deprivation) to produce an overall relative measure of deprivation.

Mental Health Diagnoses

Data extracted from LDN included severe mental illness (SMI) status using Quality and Outcomes Framework (QOF) criteria which is an annual reward and incentive programme for all GP practices in England [10]. Data extracted from CRIS included ICD-10 diagnosis consistent with the same SMI diagnoses: F20; F22; F23; F25; F28; F29 (schizophrenia and related disorders, schizoaffective disorders and delusional disorders); F30 ; F31 (mania and bipolar affective disorders); F32.3, F33.3 (psychotic depression). Thus a subgroup of the exposed cohort was defined as SMI if they were part of the SMI register on LDN or had an ICD-10 diagnosis of SMI recorded on CRIS.

Data extracted from LDN also included those on the Depression register, which is defined as patients who have an unresolved record of depression in their clinical record [10].

Physical Health Variables derived from Read codes

The following Read Codes were extracted if present during study window:

Body Mass Index (BMI): Women were defined as overweight during the study window if they had BMI value recorded of over 25.0 or had Read codes in register associated with BMI above 25 or obesity. Women were defined as underweight when they had a BMI <18.5 or Read codes in their register consistent with an anorexia nervosa diagnosis. When several BMI values were available we used the most recent.

Smoking: Read codes for current smoker

High risk alcohol use: AUDIT-C questionnaire score >=5 or Read codes for hazardous, harmful alcohol use or alcohol dependence

Drug use: Read codes for substance misuse

Folic acid prescription: Extracted via Dictionary of medicines and devices (DMD) codes [11] for all folic acid formulary preparations

Vitamin D deficiency: Read codes for vitamin D deficiency

Emergency and Long acting reversible contraception (LARC): DMD and relevant Read codes for LARC and emergency contraception prescriptions.

Physical health diagnoses: Read codes for diagnoses including asthma, hypertension, diabetes, epilepsy, HIV, hepatitis B and C, endometriosis and Polycystic Ovarian Syndrome (PCOS).

Psychiatric drugs: DMD codes on LDN and prescriptions from CRIS for Valproate, Lithium, antidepressant and antipsychotic medication.

Health care use: Number of LDN recorded primary care consultations included consultations with a doctor or nurse as face-to-face GP appointments, emergency consultations, home visits, out of

For peer review only

hours or walk in clinics. Number of CRIS recorded psychiatric inpatient days and number of face-to-face attended community contacts.

Mortality: Number of deaths was ascertained by calculating number of people with a date of death extracted from LDN at the time of data extraction.

Data Analysis

Data were analysed in Stata 15.0 for Mac after cleaning. Descriptive statistics were used to look for differences in demographic factors between exposed cohort and unexposed cohort.

Univariate and multivariate logistic and negative binominal regression analyses (adjusting for *a priori* determined confounders of IMD and ethnicity) were used to examine the rate of risk factors, diagnoses and health services contacts across groups (controls vs women in contact with mental health services) and a subgroup analysis within the exposed group (SMI vs non SMI diagnoses). A complete case analysis was also conducted to explore disparities in the outcomes above between different ethnic groups in primary care with adjustment for IMD, SMI diagnoses and age.

Patient and public involvement

The authors had no direct contact information of the study participants because anonymised clinical data were used in accordance with strict confidentiality guidelines. All patients have the choice to opt-out of their anonymised data being used. CRIS and LDN were developed with extensive service user involvement and adheres to strict governance frameworks managed by service users. No patient was involved in developing the hypothesis, research questions, plans for the study's design or writing of the results. Results will be disseminated to local public health structures including Lambeth Health Watch.

RESULTS

There were 3,817 women from LDN who were in contact with mental health services (Figure 1). These women were more likely to be from Black ethnic groups [OR 1.48 (1.31-1.67) p<0.01] and live in areas of higher deprivation [OR 1.01 (1.01- 1.02) p<0.001] compared to the rest of the LDN cohort. 688 (18.0%) of the women in contact with mental care had a recorded SMI diagnosis; 420 (71.7%) of these women had recorded diagnoses of SMI on both LDN and CRIS; 102 (14.8%) women had a SMI diagnosis recorded on CRIS only and 166 women (24.1%) had SMI diagnosis recorded on LDN only. Subgroup analysis showed that women with SMI were more likely to be older and live in areas of higher deprivation (table 1). Women with SMI diagnosis recorded on LDN but not on CRIS had higher odds of substance misuse but not multi-morbidity.

Missing data

Around 17.4% (n = 2,535) of women in the control group and 323 (8.5%) women in contact with mental health services had missing data on ethnicity. 621 (4.3%) of women in the control group and 104 women (2.7%) in women in contact with mental health (MH) services had missing data on IMD (table 1).

Missing data on ethnicity was associated with not being in contact with MH services, older age, living in areas of less deprivation and being a non-smoker; missing data on IMD was associated with younger age and Asian ethnicity.

For peer review only

532 (13.9%) women in contact with MH services and 4,582 (31.5%) in the comparison group had missing data on BMI; this was associated with not being in contact with MH services, older age and being of an ethnicity other than White British.

Table 1. Characteristics of women of reproductive age in our sample: by contact with mental health services.

	**Primary care Controls** (n=14,532)		**In contact with local MH services** (n=3,817)		
		Missing	no SMI (n=3,129)	SMI diagnoses (n=688)	Missing
**Age**		0 (0.0%)			0 (0.0%)
15-19	3,030 (20.9%)		762 (25.7%)	111 (16.1%)	
20-24	3,430 (23.6%)		718 (24.2%)	128 (18.6%)	
25-29	2,706 (18.6%)		541 (18.2%)	115 (16.7%)	
30-34	2, 312 (15.9%)		442 (14.9%)	130 (18.9%)	
35-40	3, 054 (21.0%)		506(17.0%)	204 (29.6%)	
**Ethnicity**		2,535(17.4%)			323 (8.5%)
White British	2,467 (20.6%)		698 (24.5%)	92 (14.3%)	
Other white	4,615 (38.5%)		736 (25.8%)	152 (23.6%)	
Black	1,392 (11.6%)		452 (15.9%)	206 (32.0%))	
Asian	830 (6.9%)		149 (5.2%)	32 (5.0%)	
Other	2, 693 (22.5%)		816 (28.6%)	161 (25.0%)	
**IMD score**		621 (4.3%)			104 (2.7%)
1st quintile	2,211 (15.9%)		561 (18.4%)	153 (23.0%)	
2nd quintile	6,615 (47.6%)		1,490 (48.9%)	325 (48.9%)	
3rd quintile	3,783 (27.2%)		791 (26.0%)	158 (23.8%)	
4th quintile	1,078 (7.8%)		168 (5.5%)	27 (4.1%)	
5th (least deprived)	224 (1.6%)		38 (1.3%)	2 (0.3%)	
**Inpatient during study period (X^2)**	-		184 (5.9%)	305 (44.3%) ***	
**Number of inpatient days**					
Median test	-		Median 0 IQR[0-0]	Median 0 IQR [0 -61] ***	
**Number of days with attended CMHT f2f contacts**					
Median test	-		Median 5 IQR [1 – 21]	Median 37 IQR[7-111]***	
**Number of primary care clinical contacts**					
Median test	Median 13 IQR[2-34]		Median 59 IQR[27-110]	Median 89 IQR[37 – 157] ***	
**Mortality**	18 (0.1%)		45 (1.4%)	15 (2.2%)	

*** p<0.001 **p <0.01 *p<0.05

Inequalities in health outcomes

Women in contact with MH services vs primary care contact only

Women in contact with mental health services had a higher prevalence of all risk factors investigated (table 2 and 3). They were more likely to have a weight in the abnormal range - BMI>25 [adj OR 1.46 (1.33-1.60) p<0.0001] or BMI< 18.5 [adj OR 2.39 (2.05-2.19) p<0.001] (table 3). They had a higher prevalence of alcohol [adj OR 10.39 (8.10- 13.33) p<0.001] and substance misuse [adj OR 39.37 (23.87-64.86) p<0.001] after adjustment for ethnicity and IMD. They had higher rates of vitamin D deficiency, emergency contraception use and termination of pregnancy.

Table 2. Prevalence of risk factors and physical health disorders by contact with MH services and SMI status.

	Controls (n=14,532)	In contact with MH services (n= 3,817)	
Total = 18,349		No SMI (n=3,129)	SMI diagnoses (n=688)
**Overweight (BMI> 25) N=13,235**	3,086 (31.0%)	963 (36.3%)	343 (54.4%)
**Underweight (BMI < 18.5) N=13,235**	556 (5.6%)	299 (11.3%)	48 (7.6%)
**Smoker**	3,010 (20.7%)	1,322 (42.3%)	333 (48.4%)
**Excessive alcohol use**	92 (0.6%)	230 (7.4%)	45 (6.4%)
**Drug use**	19 (0.1%)	166 (5.3%)	38 (5.5%)
**Vitamin D deficiency**	469 (3.2%)	324 (10.4%)	84 (12.2%)
**Vitamin D prescription**	632 (4.4%)	525 (16.8%)	126 (18.3%)
**Folate prescription**	294 (2.0%)	230 (7.4%)	122 (17.7%)
**LARC Use**	655 (4.5%)	280 (8.9%)	50 (7.3%)
**Emergency Contraception**	374 (2.6%)	239 (7.6%)	55 (8.0%)
**TOP**	153 (1.0%)	89 (2.8%)	11 (1.6%)
**Psychiatric medication prescribed**			
**Valproate**	12 (0.1%)	29 (0.9%)	129 (18.8%)
**Antidepressants**	1,467 (10.1%)	2,045 (65.4%)	470 (68.3%)
**Antipsychotics**	51 (0.4%)	302 (9.7%)	536 (77.9%)
**Physical Health diagnoses**			
**Asthma**	851 (5.9%)	427 (13.7%)	91 (13.2%)
**Diabetes**	174 (1.2%)	93 (3.0%)	53 (7.7%)
**Hypertension**	258 (1.8%)	91 (3.0%)	45 (6.5%)
**Cardiovascular disease**	27 (0.2%)	7 (0.2%)	5 (0.7%)
**Epilepsy**	50 (0.3%)	77 (2.5%)	18 (2.6%)
**Endometriosis**	156 (1.1%)	73 (2.3%)	9 (1.3%)

For peer review only

**PCOS**	370 (2.6%)	174 (5.6%)	27 (3.9%)
**HIV**	19 (0.1%)	29 (0.9%)	4 (0.6%)
**Hepatitis B/C**	26 (0.2%)	48 (1.5%)	12 (1.7%)
**Multi-morbidity (2 or more long term conditions)**			
**Physical**	206 (1.4%)	269 (8.6%)	74 (10.8%)
**Mental and Physical**	395 (2.7%)	660 (21.1%)	428 (62.2%)-

Women in contact with mental health services had a higher prevalence of physical multi-morbidity (two or more physical health disorders) [9.0% vs 1.4%; adj OR 2.93 (2.37 – 3.63) p<0.001]. They had a particularly high prevalence of epilepsy [adj OR 5.73 (4.00- 8.20) p <0.001], HIV [adj OR 4.77 (2.66- 8.56) p<0.001] and chronic hepatitis [adj OR 8.24 (5.12- 13.24) p<0.001] (table 2 and 3).

Table 3. Association between contact with local MH services and risk factors, diagnoses and clinical contacts.

	**In contact with MH services Univariate logistical regression OR, 95% CI** (n=18,349)	**Multivariate logistic regression Adjusted model for Ethnicity and IMD Adj OR , 95% CI** (n=14,980)
**Overweight**	1.64 (1.50-1.78) *** n=13,235	1.46 (1.33 -1.60)*** n= 12,116
**Underweight**	2.41 (2.09-2.79)*** n=13,235	2.46(2.09-2.89)*** n=12,116
**Smoking**	2.93 (2.72-3.16)***	2.78(2.56-3.02)***
**Alcohol abuse**	12.19 (9.60-15.47) ***	10.39 (8.10- 13.33)***
**Drug use**	43.13 (26.91- 69.11)***	39.35 (23.87-64.86)***
**Folate prescription**	4.92 (4.19- 5.77)***	4.05 (3.42-4.80)***
**Vitamin D deficiency**	3.58 (3.12-4.11)***	2.92 (2.52- 3.39)***
**Vitamin D prescription**	4.52 (4.03- 5.08)***	3.71 (3.27-4.21)***
**LARC**	2.01 (1.75- 2.30) ***	1.67 (1.44- 1.94)***
**Emergency contraception**	3.16 (2.70- 3.70) ***	2.70 (2.28 -3.19)***
**TOP**	2.52 (1.96- 3.26) ***	2.10 (1.60- 2.76)***
Physical health diagnoses		**Adjusted model for Ethnicity and IMD Adj OR , 95% CI** (n=11,989)
**Asthma**	2.52 (2.25- 2.83)***	2.08 (1.84- 2.35)***
**Diabetes**	3.28 (2.62- 4.10) ***	2.55 (2.01- 3.24) ***
**Hypertension**	2.07 (1.68- 2.56) ***	1.39 (1.11 - 1.74) *
**Epilepsy**	7.39 (5.24- 10.43) ***	5.73 (4.00 - 8.20)***
**PCOS**	2.13 (1.78 -2.53) ***	1.75 (1.31 - 2.32)***
**Endometriosis**	2.02 (1.54- 2.65) ***	1.87 (1.55- 2.25)***
**HIV**	6.66 (3.78- 11.73)***	4.77 (2.66 – 8.56)**
**Hepatitis B/C**	8.91 (5.62 -14.14)***	8.24 (5.12 – 13.24)***
Mortality	12.88 (7.60- 21.83)***	16.10 (8.37- 30.91)***

For peer review only

Multi-morbidity		
**Physical**	4.09 (3.34- 5.00)***	2.93 (2.37 – 3.63)***
**Mental and Physical**	14.3 (12.6 – 16.1)***	11.7 (10.3 – 13.3)***
Health contacts	**Univariate negative binominal regression IRR, 95% CI** (n=18, 165)	**Adjusted model for SMI, age, IMD, ethnicity and Diabetes Adj IRR, 95%CI** (n=14,841)
**GP consultations**	3.47 (3.31- 3.64)***	2.92 (2.80-3.05)***

*** p<0.001 **p <0.01 *p<0.05

Only 28.4% of all patients prescribed valproate had a folic acid prescription issued in primary care. Similarly whereas women on valproate were more likely to be given LARC in primary care [OR 2.27 (1.40-3.68) p<0.001], only 10.6% of women on valproate were on LARC.

Women in contact with mental health services had a higher number of face-to-face clinical consultations in primary care during study period [adj IRR 2.74 (2.62-2.87) p<0.001] after adjusting for socio-demographic factors, SMI and a diabetes diagnosis. They were approximately sixteen times more likely to die [adj OR 16.10 (8.37 – 30.91) p<0.001] after adjusting for IMD and ethnicity and most deaths were among women without a SMI diagnosis (table 1 and table 3).

Subgroup analysis: SMI vs non SMI diagnoses in woman in contact with mental health services

Women with SMI were more likely to be overweight [adj OR 1.80 (1.48 -2.19) p<0.001] ; smokers [adj OR 1.41 (1.18- 1.69) p<0.001]; have a diagnosis of diabetes [adj OR 2.31(1.58- 3.36) p<0.001] and hypertension [adj OR 1.71 (1.15- 2.56) p<0.001] than other women in contact with mental health services with no SMI diagnoses after adjusting for IMD and ethnicity (supplementary file 1). They were also more likely to have a higher number of attended community mental health face to face appointments, inpatient days in mental health services, as well as GP face to face consultations during the study period.

Racial and Ethnic disparities

Mental Health

Across cases and controls with data on ethnicity (n=15, 491), there were significant discrepancies in diagnosis of depression in primary care. Fifteen percent of the whole sample (n=2,688) was on the primary care depression register. Women from White Other [adj OR 0.49 (0.44-0.56) p<0.001], Asian [adj OR 0.53 (0.43-0.65) p<0.001] and Black groups [adj OR 0.67 (0.57-0.78) p <0.001] were less likely to be diagnosed with depression in primary care compared to White British women after adjustment for age and IMD. Conversely, Black women were more likely to have a SMI diagnosis [adj OR 2.79 (2.13- 3.64) p<0.001] (table 4 and 5).

For peer review only

Table 4. Prevalence of mental and physical health diagnoses, risk factors and clinical contacts by ethnicity in total sample.

Total (n= 15, 491)	White British (n= 3,357, 21.1%)	White other (n=5,503, 35.5%)	Black (n= 2,050, 13.2 %)	Asian (n=1,011, 6.5%)	Other (n=3,670, 23.7%)
SMI	92 (2.8%)	152 (2.8%)	206 (10.1%)	32 (3.2%)	161 (4.4%)
Depression	660 (20.3%)	659 (12.0%)	361 (17.6%)	126 (12.5%)	748 (20.4%)
Asthma	320 (9.8%)	303 (5.5%)	193 (9.4%)	73 (7.2%)	411 (11.2%)
Diabetes	35 (1.1%)	62 (1.1%)	111 (5.4%)	32 (3.2%)	57 (1.6%)
Hypertension	34 (1.0%)	67 (1.2%)	192 (9.4%)	20 (2.0%)	61 (1.7%)
Epilepsy	32 (1.0%)	23 (0.4%)	24 (1.8%)	8 (0.8%)	50 (1.4%)
Endometriosis	40 (1.2%)	58 (1.1%)	44 (2.2%)	15 (1.5%)	69 (1.9%)
PCOS	131 (4.0%)	154 (2.8%)	62 (3.0%)	32 (3.2%)	142 (3.9%)
HIV	2 (0.1%)	8 (0.2%)	35 (1.7%)	0 (0.0%)	6 (0.2%)
Hepatitis B/C	17 (0.5%)	27 (0.5%)	15 (0.7%)	6 (0.6%)	21 (0.6%)
Overweight (BMI>25) n=12,353	730 (26.0%)	1,183(28.0%)	1,012 (62.2%)	206 (27.1%)	989 (32.3%)
Underweight BMI<18.5 N=12,353	172 (6.1%)	295 (7.0%)	88 (5.4%)	82 (10.8%)	209 (6.8%)
Smoker	921 (28.3%)	1,685 (30.6%)	414 (20.2%)	165 (16.3%)	1,157(31.5%)
Alcohol abuse	107 (3.3%)	83 (1.5%)	33 (1.7%)	9 (1.1%)	114(2.8%)
Drug use	59 (1.8%)	58 (1.1%)	44 (2.2%)	15 (1.5%)	69 (1.9%)
Vit D deficiency	109 (3.4%)	167 (3.0%)	269 (13.1%)	94 (9.3%)	189 (5.2%)
LARC use	265 (8.1%)	264 (4.8%)	119 (5.8%)	31 (3.1%)	238 (6.5%)
EHC	132 (4.1%)	150 (2.7%)	139 (6.8%)	26 (2.6%)	170 (4.6%)
TOP	51 (1.6%)	51 (0.9%)	48 (2.3%)	23 (2.3%)	63 (1.7%)
Antipsychotic use	135 (4.1%)	181 (3.3%)	237 (11.6%)	44 (4.4%)	227 (6.2%)
Antidepressant use	903 (27.7%)	981 (17.8%)	596 (29.1%)	178 (17.6%)	1,035 (28.2%)
Physical multi-morbidity	68 (2.1%)	68 (1.2%)	130 (6.3%)	26 (2.6%)	81 (2.2%)
Physical and mental multi-morbidity	286 (8.8%)	286 (5.2%)	342 (16.7%)	72 (7.1%)	419 (11.4%)
Number of contacts in primary care	Median 26 IQR[12-54]	Median 14 IQR [4-38]	Median 43 IQR [12-101]	Median 18 IQR[5 -53]	Median 28 IQR [11-60]
Number of contacts in CMHT	Median 9 IQR[1- 30]	Median 4 IQR[1- 25]	Median 10 IQR[1- 43]	Median 5 IQR[1 – 24]	Median 7 IQR[1- 32]
Mortality	21 (0.6%)	15 (0.3%)	20 (1.0%)	5 (0.5%)	8 (0.2%)

For peer review only

Table 5. Association between ethnicity, risk factors, health diagnoses and clinical contacts.

Base (White British)	**Multivariate logistical regressions** Adj Odds Ratio (OR) ; 95% CI **Model adjusted for IMD and age (ordinal) (n=14, 980)**			
	**White other**	**Black**	**Asian**	**Other**
**SMI**	0.88 (0.64 – 1.09)	2.79 (2.13- 3.64)***	1.07 (0.71-1.61)	1.42 (1.09-1.85)**
**Depression**	0.49 (0.44-0.56)***	0.67 (0.57-0.78)***	0.53(0.43-0.65)***	0.95 (0.84 – 1.07)
**Model adjusted for IMD, SMI and age (n=14, 980)**				
**Overweight**	0.97 (0.86- 1.08) (n= 12, 116)	3.47 (3.00-4.01)*** (n= 12, 116)	0.96 (0.79 – 1.17) (n= 12,116)	1.24(1.10-1.40)*** (n= 12, 116)
**Underweight**	1.22 (0.99 - 1.49) (n= 11,989)	1.79 (1.35-2.39)*** (n= 12, 116)	1.96(1.44 -2.63)*** (n= 12, 116)	1.28(1.03-1.59)* (n= 12, 116)
**Smoking**	1.10(0.99 - 1.21)	0.53(0.46-0.61)***	0.47 (0.39-0.57)***	1.12(1.01-1.24)*
**Alcohol abuse**	0.38 (0.28 - 0.51)***	0.32 (0.21-0.49)***	0.26 (0.14-0.51)***	0.84 (0.64-1.10)
**Drug use**	0.46 (0.32-0.69)***	0.50 (0.32-0.79)**	0.28 (0.12-0.66)**	0.71 (0.49-1.03)
**Vitamin D Deficiency**	0.79 (0.62-1.02)	3.03 (2.28- 3.87)***	2.72 (2.04 – 3.64)***	1.40 (1.09-1.78)***
**LARC**	0.56 (0.46-0.67)***	0.70 (0.56-0.89)**	0.36 (0.24-0.54)***	0.77(0.64-0.92)**
**Emergency contraception**	0.72 (0.56-0.92)**	1.86 (1.43-2.42)***	0.64 (0.41-1.00)	1.19 (0.94-1.52)
**TOP**	0.60 (0.40- 0.90)*	1.68 (1.10 -2.56)*	1.57 (0.94 -2.65)	1.14 (0.78-1.66)
**Antidepressant**	0.52 (0.46-0.58)***	0.75 (0.65-0.86)***	0.51(0.42 -0.62)***	0.94 (0.84-1.05)
**Antipsychotic**	0.66 (0.49 –0.89)**	1.48 (1.08-2.04)*	0.91(0.57-1.45)	1.32 (0.99-1.76)
**Valproate**	0.71 (0.41-1.24)	1.29 (0.76-2.26)	0.69 (0.27 – 1.75)	1.25 (0.74- 2.11)
**Lithium**	0.68 (0.36-1.28)	0.65 (0.35-1.23)	0.69 (0.24- 1.96)	0.78 (0.41-1.45)
**Model adjusted for IMD, SMI, valproate use and age (n=14, 841)**				
**Folate prescription**	0.86(0.64 -1.14)	2.45 (1.84-3.25)***	1.67 (1.14-2.43)***	1.37 (1.03-1.82) *
**Model adjusted for IMD, SMI and age (n= 11,989)**				
**Asthma**	0.53(0.45-0.63)***	0.89 (0.73 – 1.09)	0.75 (0.57-0.98) *	1.14(0.97-1.33)
**Diabetes**	0.76 (0.50-1.16)	2.26 (1.51 -3.40)***	2.20 (1.34-3.60)**	1.05(0.68- 1.62))
**Hypertension**	0.83 (0.54- 1.27)	3.95 (2.67 – 5.85)***	1.33 (0.75- 2.35)	1.15 (0.74 – 1.77)
**Epilepsy**	0.36(0.21-0.62)***	0.63(0.38-1.20)	0.69 (0.32- 1.52)	1.17 (0.74- 1.84)
**PCOS**	0.80 (0.63- 1.02)	0.96 (0.69- 1.32)	0.97 (0.65 -1.44)	1.05 (0.82-1.34)
**Endometriosis**	0.80 (0.53-1.20)	1.46 (0.92 -2.32)	1.00 (0.73-2.32)	1.43 (0.96-2.13)
**Multimorbidity**	**Model adjusted for IMD and age (n= 14, 980)**			
**Physical**	0.49 (0.34-0.69)***	1.94 (1.41-2.68)***	1.05 (0.66 – 1.68)	0.89 (0.64-1.24)
**Mental and Physical**	0.98 (0.41-0.58)***	1.40 (1.17-1.67)***	0.71 (0.54 -0.94)*	1.19 (1.01-1.40) *
**Health care contacts**	**Multivariate Negative Binominal Regression Model adjusted for IMD, SMI and age adj IRR , 95% CI**			
**GP Consultations**	0.63 (0.60-0.66)*** (n=14,980)	1.26 (1.18-1.35)*** (n=14,980)	0.85 (0.78-0.93)*** (n=14,980)	1.09(0.95 -1.06) (n=14,980)
**CMHT f2f contacts**	0.59 (0.50-0.68)*** (n=3,399)	0.80 (0.68-0.95)* (n=3,399)	0.58(0.44 - 0.75)*** (n=3,399)	0.81(0.70-0.94)** (n=3,399)
**Inpatients days**	0.88 (0.46 -1. 67) (n=3,494)	0.75 (0.36 -1.56) (n=3,494)	0.92 (0.33- 2.53) (n=3,494)	1.75 (0.97 -3.15) (n=3,494)

For peer review only

Risk factors

Black women [adj OR 3.47 (3.00 – 4.01) p <0.001] and women from Mixed/Other ethnicities [adj OR 1.24 (1.10 -1.40) p<0.001] were significantly more likely to be overweight after adjusting for SMI status, IMD and age and Black, Asian and women from Mixed/ Other ethnicities were also more likely to have a BMI below 18.5 compared to White British women (table 5 and supplementary file 2).

Both Black and Asian women were less likely to smoke, use alcohol excessively or misuse substances than White British women.

Black [adj OR 3.03(2.38- 3.87) p<0.001], Asian [adj OR 2.72 (2.04 – 3.64) p<0.001] and women from mixed and other ethnicities [adj OR 1.40 (1.09 -1.78) p<0.001] were more likely to be Vitamin D deficient. They were also more likely to be prescribed folic acid after adjustment for SMI, valproate use and age (table 5).

LARC prescription was low in our sample with less than 10% of women obtaining this method in primary care. Women from all ethnic minority groups were less likely to be prescribed or administered LARC in primary care compared to White British women, uptake was particularly low in Asian women [adj OR 0.36 (0.24-0.54) p<0.001]. Women from Black ethnic groups were also more likely to visit GP for emergency contraception [adj OR 1.64(1.27-2.12) p<0.001] and termination of pregnancy [adj OR 1.68 (1.10-2.56) p=0.016].

Psychiatric Medication

Black, Asian and women from White Other ethnic groups were less likely to be prescribed antidepressant medication in primary care compared to White British women. Whereas Black women were more likely to be prescribed antipsychotic medication after adjustment for SMI diagnoses, IMD and age.

Physical health diagnoses

Asthma was the most prevalent (7.5% of total sample) physical health diagnosis in all ethnic groups except in Black women where hypertension was the most prevalent diagnosis (9.4% of all Black women) (table 4).

Black women were almost four times more likely to be diagnosed with hypertension than White British women [adj OR 3.95 (2.67 – 5.85) p<0.001] .Diabetes was also more prevalent among Black [adj OR 2.26 (1.51- 3.40) p<0.001] and Asian women [adj OR 2.20 (1.34-3.60) p<0.001] compared to White British women (table 5).

The prevalence of PCOS and endometriosis was 3.1% and 1.3% of total sample respectively and there were no differences among different ethnic groups.

The numbers of women with HIV and hepatitis B/C in our sample were too small to conduct multivariate logistical regression analyses but the prevalence of HIV was disproportionally higher in Black women (1.7%) compared to whole sample prevalence (0.3%) (table 4).

For peer review only

Overall, Black women had higher rates of physical multi-morbidity compared to White British women [prevalence 6.3% vs 2.1%; adj OR 1.94 (1.41 –2.68) p<0.001] (table 4 and 5).

Health care contacts

Black women had a higher number of face-to-face primary care appointments during the study period than women from other ethnic groups after adjustment for IMD, SMI diagnoses and age [adj IRR 1.26 (1.18-1.35) p<0.001] (table 5).

After adjustment for SMI and IMD results showed that all ethnic minority groups had less community mental health face-to-face contacts compared to White British women.

Of the 15,491 women with data on ethnicity there were 69 (0.5%) deaths. The numbers were too small to perform logistical regression per ethnic group but there were significant differences with Black women having the higher risk of death (n =20, 1.0% fisher exact test p<0.001] (table 4).

DISCUSSION

Principal findings

In this study using a primary care- secondary mental health care clinical data linkage of an ethnically diverse and deprived inner London borough, we found striking differences in risk factors, multi-morbidity and health service contacts by ethnicity and mental health service use for women of reproductive age. Women in contact with mental health services had higher prevalence of all physical health diagnoses and risk factors studied including a BMI outside the healthy range, smoking, alcohol, and substance misuse as well as micronutrient deficiencies. They also had higher rates of emergency contraception use and termination of pregnancy stressing a greater unmet need for contraception. Similarly to previous studies, we found the risk of HIV and chronic hepatitis [12] to be greatly elevated compared to controls. Adjusting for hypothesized exploratory factors including ethnicity, deprivation and SMI diagnosis only partly explained inequalities. The risk of all-cause mortality in this group was 16 times higher compared to controls and, notably, women without an SMI diagnosis were also at increased risk of death. Black women and women in contact with mental health services had a higher number of face-to-face clinical contacts in primary care so inequalities cannot be exclusively explained by lack of access.

Comparison with other studies

Our study adds to growing literature of inequalities in mental and physical health outcomes for women of ethnic minority groups in the UK. Our results echoes studies during the perinatal period which found women from ethnic minority groups are less likely to be diagnosed and receive treatment for depression in primary care [13] and paradoxically Black women are more likely to have an SMI diagnosis [14]. We found that Black and Asian women were less likely to engage in some risk behaviours, such as smoking or drinking alcohol excessively compared to White British but were more likely to have unhealthy weight. Black women were also more likely to be prescribed antipsychotics irrespective of diagnosis. There were also inequalities in sexual and reproductive health and women from ethnic minority groups had decreased uptake of LARC in primary care. Consistent with previous national survey data [15], Black women had higher use of emergency contraception than White British women. We also confirmed that Black women were at disproportionally high risk of hypertension compared to White British women [16].

For peer review only

Strengths and Weaknesses

To our knowledge this is one of the few studies investigating multi-morbidity in non-pregnant women of reproductive age at the population level and highlighting disparities by ethnicity and mental ill-health, with a large sample size and near complete coverage of primary care data in a London borough. Limitations include the exclusion of highly mobile populations not in contact with the same practice for 2 years, who may be at higher risk of poor health and under recording of risk factors and diagnoses (potentially due to clinicians' unconscious biases and lack of incentives to record risk factors in patients without SMI). Previous studies have found differences in multi-morbidity within the broader ethnic groups defined in this study (e.g. Black Caribbean and Black African) [17] but further disaggregation was not possible due to small cells. Our data on use of contraception must also be interpreted with caution, as these are patterns of use and access in primary care whereas there are alternative sources of these services in community sexual health clinics and pharmacies. National data has shown that women living in urban areas and from ethnic minority groups are more likely to visit sexual health clinics compared to White British women but primary care remains the main provider of contraception across population groups and inequalities persist despite the source of services [18]. Finally, we also did not have cause specific mortality and further research is required to elucidate this.

Policy implications

Our study highlights that striking differences in multimorbidity are present in ethnic minority groups with and without mental illness at the preconception stage which may partly explain inequalities in pregnancy outcomes and mortality for Black and Asian women during the perinatal period in the UK [19]. These differences suggest that there are major structural inequalities in healthcare provision in the UK. There is evidence that common mental disorders are underrecognized by primary care clinicians in women of ethnic minority groups, which has repercussions across the health system as to how women´s needs are properly assessed and prioritized by other services including maternity services. The dominance of medical model of mental illness and organization around diagnostic criteria can also lead to inattention to people´s lived experience and individual contexts, neglecting cultural differences in expressing distress and potentially leading to further re-traumatisation [20] .

As part of the COVID-19 response, NHS (National Health Service) England has issued guidance setting urgent priorities to tackle health inequalities, particularly around maternal health [21] . There is a drive to understand the needs of local populations – its health outcomes and community assets and use this understanding to plan co-production activity to design interventions to improve equity for women and babies. There is also a push to promote personalised care and support plans as well as ensure women from ethnic minority groups are represented in peer and lay roles within local health and wellbeing programmes. Under the NHS Long Term Plan, maternal medicine networks will be established so that by March 2024 every woman in England with medical problems has access to specialist advice and care. In addition, maternal mental health services are being developed to bring together maternity, psychology and reproductive health services for women who develop moderate–severe mental ill health from loss or trauma due to their maternity experience. These services are a substantial step forward although there is a risk they will focus on perinatal trauma without recognition of how cumulative adversity along the life course, including racism, influences women´s experiences of health services. Without embracing the wider complexity, the needs of women experiencing multiple intersecting disadvantages will continue to go unknowledge and fall between the gaps.

Our results also challenge the perspective that regular monitoring of physical health in primary care should be exclusively encouraged in people with a SMI diagnosis and indicate that there are mental

For peer review only

and physical health needs of women in contact with mental health services that are not being met by the current model of service provision. Our study emphasizes the need for a more holistic approach to health promotion for women in contact with mental health services, expanding the remit beyond cardiovascular disease prevention into sexual and reproductive health, including avoidance of drugs with teratogenic potential and addressing micronutrient deficiencies and substance misuse.

CONCLUSIONS

There are striking inequalities in risk factor profile and health outcomes including mortality for women of reproductive age in contact with mental health services and those of ethnic minority groups emphasising a need of culturally centered integrative models of care. More attention should be focused on identifying missed opportunities to intervene across primary and secondary mental health services and closer attention should be given to how cumulative adversity along the life course, including experiences of racism, impacts on women´s access to and experiences of health services.

Statements

Contributor and guarantor information:
RC conceived the research question and designed the study analysis plan with input from LMH and SLH. MP, AJ and MB performed data extraction. RC and SD analysed the data. RC wrote the first draft of the manuscript. LMH, SLH, MA and SD critically revised it. All authors approved the final version of the manuscript. The corresponding author attests that all listed authors meet authorship criteria and that no others meeting the criteria have been omitted.
The guarantor is RC, who affirms that the manuscript is an honest, accurate, and transparent account of the study being reported, that no important aspects of the study have been omitted, and that any discrepancies from the study as originally planned have been explained.

Acknowledgements

This work uses data provided by patients and collected by the NHS as part of their care and support . We are grateful for all the support received from the **National Institute for Health Research (NIHR)** BRC Maudsley clinical informatics teams throughout this work,

Financial support

This project has been funded by the Closing the Gap network. Closing the Gap is funded by UK Research and Innovation and their support is gratefully acknowledged (Grant reference: ES/S004459/1). The funding source had no role in project design, data analysis or writing the manuscript. This project was possible due to access to the data at the **NIHR Maudsley Biomedical Research Centre at South London and Maudsley NHS Foundation Trust and King's College London.** RC and SD receive salary support from NIHR as clinical academics (no award numbers). SLH is part-funded by the NIHR Biomedical Research Centre at South London and Maudsley NHS Foundation Trust and supported by the ESRC Centre for Society and Mental Health at King's College London (ESRC Reference: ES/S012567/1). SLH also receives funding from Guy's and St Thomas' Charity and Wellcome Trust (no award number). LMH is a NIHR Principal Investigator and receives funding from **NIHR Maudsley Biomedical Research Centre,** NIHR Applied Research Collaboration South London and UKRI funded Network on Violence, Abuse and Mental Health (no award numbers) . Any views expressed here are those of the project investigators and do

For peer review only

not necessarily represent the views of the Closing the Gap network, UKRI, NIHR, ESRC, MRC, Wellcome Trust, NHS or King's College London.

Conflicts of Interest

All authors have completed a ICMJE disclosure form http://www.icmje.org/disclosure-of-interest/ and declare financial support from Closing the Gap Network and NIHR for the submitted work; SLH receives consulting fees from the NHS Race and Health Observatory; no financial relationships with any organisations that might have an interest in the submitted work in the previous three years; All authors declare no other relationships or activities that could appear to have influenced the submitted work.

Ethical Standards and data sharing statement

Clinical Record Interactive Search (CRIS) was established in 2008 and approved by the Oxfordshire Research Ethics Committee in 2008 (reference 18/SC/0372). Approval for linkage with LDN was granted by Lambeth Clinical Commissioning Group and Information Governance Steering Group. The anonymised dataset that underlie the results in this article is only available on request to investigators whose proposed use of data has been approved by CRIS Oversight Committee and Lambeth Clinical Commissioning Group and Information Governance Steering Group. Complete list of codes used to identify study population and outcomes can be requested from the corresponding author (Raquel.catalao@kcl.ac.uk).

License for publication:
The Corresponding Author has the right to grant on behalf of all authors and does grant on behalf of all authors, a worldwide licence to the Publishers and its licensees in perpetuity, in all forms, formats and media (whether known now or created in the future), to i) publish, reproduce, distribute, display and store the Contribution, ii) translate the Contribution into other languages, create adaptations, reprints, include within collections and create summaries, extracts and/or, abstracts of the Contribution, iii) create any other derivative work(s) based on the Contribution, iv) to exploit all subsidiary rights in the Contribution, v) the inclusion of electronic links from the Contribution to third party material where-ever it may be located; and, vi) licence any third party to do any or all of the above.

Provenance and peer review: Not commissioned; externally peer reviewed.

List of Abbreviations:
BMI: Body Mass Index
CRIS: Clinical Records Interaction Search
DMD: Dictionary of Medicines and Devices
GP: General Practitioner
IMD: Index of Multiple Deprivation
LARC: Long Acting Reversible Contraception
LDN: Lambeth DataNet
MH: Mental Health
NHS: National Health Service
PCOS: Polycystic Ovary Syndrome
QOF: Quality Outcome Framework
SLaM: South London and the Maudsley NHS Foundation Trust
SMI: Severe Mental Illness

For peer review only

References:

1 Thornicroft G. Premature death among people with mental illness. BMJ. 2013;**346**. doi:10.1136/bmj.f2969

2 Stein A, Pearson RM, Goodman SH, *et al.* Effects of perinatal mental disorders on the fetus and child. *Lancet* 2014;**384**:1800–19. doi:10.1016/S0140-6736(14)61277-0

3 Knight M, Bunch K, Kenyon S, *et al.* A national population-based cohort study to investigate inequalities in maternal mortality in the United Kingdom, 2009-17. *Paediatr Perinat Epidemiol* 2020;**34**:392–8. doi:10.1111/ppe.12640

4 Office of National Statistics. 2011 Census: KS201 UK Ethnic group, local authorities in the United Kingdom. 2011. https://www.ons.gov.uk/peoplepopulationandcommunity/populationandmigration/populationestimates/datasets/2011censuskeystatisticsandquickstatisticsforlocalauthoritiesintheunitedkingdompart1 (accessed 1 Nov 2021)

5 Lambeth.gov.uk. Demography factsheet. 2017.https://www.lambeth.gov.uk/sites/default/files/ssh-demography-factsheet-2017.pdf (accessed 1 Nov 2021)

6 GOV.UK. English Indices of Deprivation 2010. 2010.https://www.gov.uk/government/statistics/english-indices-of-deprivation-2010 (accessed 1st November 2021)

7 NHS Digital. Read Codes - NHS Digital. https://digital.nhs.uk/services/terminology-and-classifications/read-codes (accessed 1 Nov 2021).

8 Stewart R, Soremekun M, Perera G, *et al.* The South London and Maudsley NHS Foundation Trust Biomedical Research Centre (SLAM BRC) case register: Development and descriptive data. *BMC Psychiatry* Published Online First: 2009. doi:10.1186/1471-244X-9-51

9 GOV.UK. English indices of deprivation 2019 - GOV.UK. https://www.gov.uk/government/statistics/english-indices-of-deprivation-2019 (accessed 1 Nov 2021).

10 NHS Digital. QOF 2019-20 | NHS Digital. https://qof.digital.nhs.uk/ (accessed 1 Nov 2021).

11 Dictionary of medicines and devices (dm+d) | NHSBSA. https://www.nhsbsa.nhs.uk/pharmacies-gp-practices-and-appliance-contractors/dictionary-medicines-and-devices-dmd (accessed 1 Nov 2021).

12 Hughes E, Bassi S, Gilbody S, *et al.* Prevalence of HIV, hepatitis B, and hepatitis C in people with severe mental illness: A systematic review and meta-analysis. *The Lancet Psychiatry* 2016;**3**:40–8. doi:10.1016/S2215-0366(15)00357-0

13 Prady SL, Pickett KE, Petherick ES, *et al.* Evaluation of ethnic disparities in detection of depression and anxiety in primary care during the maternal period: Combined analysis of routine and cohort data. *Br J Psychiatry* 2016;**208**:453–61. doi:10.1192/bjp.bp.114.158832

14 Abel KM, Hope H, Swift E, *et al.* Prevalence of maternal mental illness among children and adolescents in the UK between 2005 and 2017: a national retrospective cohort analysis. *Lancet Public Heal* 2019;**4**:e291–300. doi:10.1016/S2468-2667(19)30059-3

15 Wayal S, Hughes G, Sonnenberg P, *et al.* Ethnic variations in sexual behaviours and sexual health markers: findings from the third British National Survey of Sexual Attitudes and Lifestyles (Natsal-3). *Lancet Public Heal* 2017;**2**:e458–72. doi:10.1016/S2468-2667(17)30159-7

16 Primatesta P, Bost L, Poulter NR. Blood pressure levels and hypertension status among

For peer review only

ethnic groups in England. *J Hum Hypertens* Published Online First: 2000. doi:10.1038/sj.jhh.1000960

17 Dorrington S, Carr E, Stevelink SAM, *et al.* Multimorbidity and fit note receipt in working-age adults with long-term health conditions. *Psychol Med* 2020;:1–10. doi:10.1017/S0033291720002937

18 French RS, Geary R, Jones K, *et al.* Where do women and men in Britain obtain contraception? Findings from the third National Survey of Sexual Attitudes and Lifestyles (Natsal-3). *BMJ Sex Reprod Heal* 2018;**44**:16–26. doi:10.1136/jfprhc-2017-101728

19 Knight M, Bunch K, Tuffnell D, *et al. Saving Lives, Improving Mothers' Care Maternal, Newborn and Infant Clinical Outcome Review Programme*. 2018.

20. Warshaw C. Limitations of the medical model in the care of battered women. *Gender Soc* 1989; **3**: 506–17.

21. NHS England » Equity and equality: Guidance for local maternity systems. https://www.england.nhs.uk/publication/equity-and-equality-guidance-for-local-maternity-systems/ (Accessed April 2022)

For peer review only

Figure 1. Exposed cohort and controls selection from Lambeth DataNet.

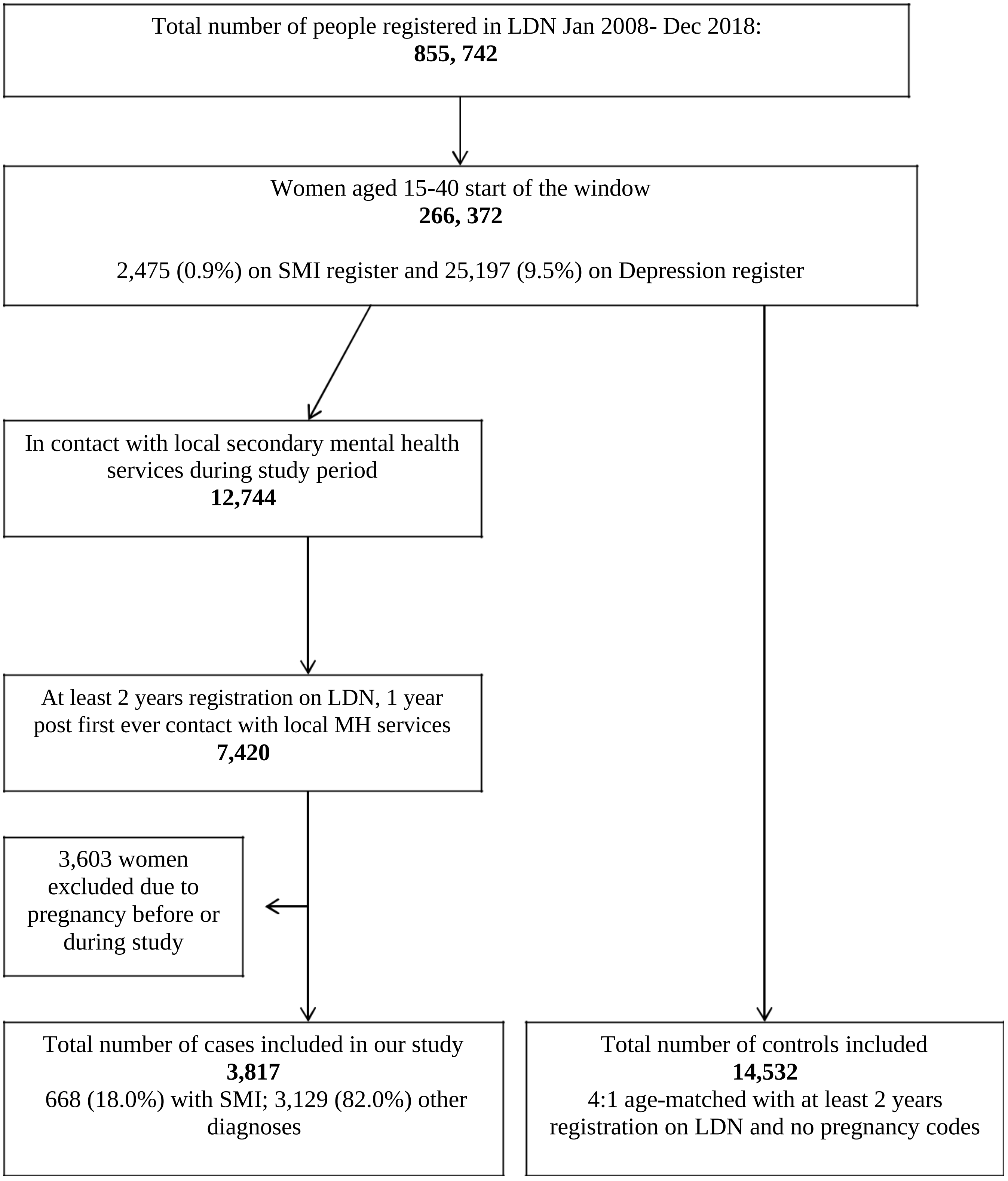

Table S1. Association between SMI and risk factors, diagnoses and health care contacts within the exposed cohort women in contact with mental health services.

	SMI<br>Univariate logistical regression<br>OR, 95% CI<br>n=3,817	Multivariate logistic regression<br>Adjusted model for ethnicity & IMD<br>Adj OR, 95% CI<br>n=3, 399
**Overweight**	2.07 (1.72-2.50)***<br>n= 3,285	1.80(1.48- 2.19) ***<br>n= 3,015
**Underweight**	0.93 (0.67- 1.31)<br>n= 3,285	0.86(0.60 - 1.22)<br>n= 3,015
**Smoking**	1.28 (1.08 - 1.51)**	1.41 (1.18 - 1.69)***
**Alcohol abuse**	0.88 (0.63 - 1.23)	0.95 (0.67 – 1.35)
**Drug use**	1.04 (0.72 - 1.50)	1.04 (0.71 - 1.52)
**Folate prescription**	2.72 (2.14 – 3.44) ***	2.20 (1.70 - 2.84) ***
**Vitamin D deficiency**	1.20 (0.93 – 1.55)	0.98 (0.75 – 1.30)
**LARC**	0.80 (0.58 - 1.09)	0.86 (0.62-1.20)
**Emergency contraception**	1.05 (0.77- 1.43)	1.02 (0.74 - 1.41)
**TOP**	0.55 (0.29 – 1.04)	0.55 (0.28 – 1.08)
Physical Health Diagnoses		
**Asthma**	0.96 (0.76 -1.23)	0.97 (0.75 - 1.26)
**Diabetes**	2.72 (1.92 – 3.86) ***	2.31 (1.58 – 3.36)***
**Hypertension**	2.28 (1.58- 3.29)***	1.71 (1.15 – 2.56) **
**Epilepsy**	1.06 (0.63 - 1.80)	1.04 (0.60 – 1.80)
**HIV**	0.62 (0.22- 1.78)	0.39 (0.13- 1.13)
**Hepatitis B/C**	1.14 (0.61- 2.16)	1.20 (0.63 – 2.31)
**PCOS**	0.69 (0.46- 1.05)	0.76 (0.49 - 1.16)
**Endometriosis**	0.55 (0.28- 1.11)	0.55 (0.27 – 1.13)
Multi-morbidity		
**Physical**	1.36 (0.9-1.9)	1.20 (0.88 – 1.82)
**Health care use**	**SMI<br>Univariate Negative binominal regression<br>IRR, 95% CI<br>n=3,817**	**Multivariate negative Binominal regression<br>Adjusted model for ethnicity& IMD<br>adj IRR, 95% CI<br>n=3, 399**
**Community MH F2F**	4.02 (3.53 – 4.57)***	4.05 (3.51 – 4.67) ***
**Inpatient MH days**	32.37 (19.50 - 53.84) ***	40.31 (21.70 – 74.88) ***
**GP consultations**	1.41 (1.30- 1.52)***	1.31 (1.21- 1.43) ***

*** p<0.001 **p <0.01 *p<0.05*

For peer review only

Table S2. Association between ethnicity, risk factors, health diagnoses and clinical contacts.

Base (White British)	Univariate logistical regressions<br>Odds Ratio (OR) 95% CI<br>**(n=15,491)**			
	**White other**	**Black**	**Asian**	**Other**
**SMI**	0.98 (0.75 -1.27)	3.84(2.99-4.95)***	1.12(0.74-1.69)	1.58(1.22-2.05)**
**Depression**	0.53(0.48-0.60)***	0.84(0.73-0.97)*	0.56(0.46-0.69)***	1.01(0.90-1.13)
**Overweight**	1.12(1.00 - 1.25) *<br>(n=12, 485)	5.00(4.37-5.73)***<br>(n=12, 485)	1.14 (0.95-1.37)<br>(n=12, 485)	1.39(1.24-1.56)***<br>(n=12, 485)
**Underweight**	1.19 (0.98-1.45)*<br>(n=12, 485)	1.85(1.41-2,43)***<br>(n=12, 485)	1.93(1.45-2.55)***<br>(n=12, 485)	1.25(1.00-1.54)*<br>(n=12, 485)
**Smoking**	1.12 (1.02-1.23)*	0.64(0.56-0.73)***	0.49(0.41-0.59)***	1.17(1.05-1.30)**
**Alcohol abuse**	0.45(0.34-0.60)***	0.54(0.37-0.79)***	0.29(0.15-0.56)***	0.97 (0.74-1.27)
**Drug use**	0.55(0.38-0.79)***	0.97 (0.64-1.47)	0.32(0.14- 0.75)*	0.86(0.59-1.23)
**Vitamin D Deficiency**	0.90(0.71-1.15)	4.36(3.46-5.49)***	2.96(2.22-3.94)***	1.57(1.23-1.99)***
**LARC**	0.57(0.48-0.68)***	0.70(0.56-0.87)***	0.36(0.24-0.52)***	0.78(0.65-0.94)**
**Emergency contraception**	0.66(0.52-0.84)***	1.72(1.35-2.20)***	0.62(0.41-0.96) *	1.15(0.91-1.45)
**TOP**	0.59(0.40-0.87)**	1.51 (1.01-2.24)*	1.46(0.89-2.41)	1.10 (0.76-1.60)
**Antidepressant**	0.56(0.51-0.63)***	1.07(0.95-1.26)	0.56(0.47-0.67)***	1.02(0.92-1.34)
**Antipsychotic**	0.79(0.67-0.99)*	3.02(2.43-3.76)***	1.05 (0.76-1.44)	1.52(1.22-1.90)***
**Valproate**	0.70(0.42- 1.19)	3.23(2.01-5.20)***	0.87 (0.37-2.02)	1.58 (0.97- 2.55)
**Lithium**	0.71 (0.39-1.28)	2.16 (1.20- 3.86)**	0.80 (0.30-2.15)	1.20 (0.67 -2.55)
**Folate prescription**	0.99 (0.75-1.30)	4.36 (3.35- 5.67)***	1.83(1.26-2.65)**	1.60(1.21-2.11)**
**Asthma**	0.53(0.45-0.63)***	0.95(0.79-1.15)	0.71(0.55-0.93) *	1.16(0.99-1.35)
**Diabetes**	1.05(0.69-1.59)	5.27(3.59-7.34)***	3.01(1.85-4.88)***	1.45(0.95-2.22)
**Hypertension**	1.17(0.77-1.77)	9.80(6.77-14.17)***	1.91 (1.10-3.34)*	1.60(1.05-2.44)*
**Epilepsy**	0.42(0.25-0.75)**	1.19(0.70-2.03)***	0.80 (0.37-1.75)	1.39 (0.89-2.17)
**PCOS**	0.69(0.53-0.87)**	0.74(0.55-1.01)	0.79 (0.53-1.16)	0.96(0.75-1.22)
**Endometriosis**	0.86 (0.57 – 1.28)	1.76(1.14-2.71)*	1.21 (0.67 – 2.20)	1.54(1.04-2.28)*
**Multimorbidity**				
**Physical**	0.59(0.42-0.82) **	3.17(2.34-4.28)***	1.24(0.78-1.96)	1.06(0.76-1.47)
**Mental and Physical**	0.57(0.48-0.67)***	2.08(1.76-2.46)***	0.80(0.61-1.04)	1.34(1.14-1.57)***
**Health care contacts**	**Univariate Negative Binominal Regression<br>IRR , 95% CI**			
**GP Consultations**	0.70(0.66-0.74)***<br>(n=15,491)	1.69(1.58-1.81)***<br>(n=15,491)	0.95(0.85-1.81)<br>(n=15,491)	1.09(1.03- 1.16)**<br>(n=15,491)
**CMHT f2f contacts**	0.68(0.58-0.80)***<br>(n=3,494)	1.52(1.28-1.81)***<br>(n=3,494)	0.80(0.61-1.04)<br>(n=3,494)	1.06(0.91-1.24)<br>(n=3,494)
**Inpatients days**	0.84 (0.43-1.62)<br>(n=3,494)	4.50(2.21-9.19)***<br>(n=3,494)	1.13(0.37-3.77)<br>(n=3,494)	1.97(1.04-3.09) *<br>(n=3,494)

For peer review only

The RECORD statement – checklist of items, extended from the STROBE statement, that should be reported in observational studies using routinely collected health data.

	Item No.	STROBE items	Location in manuscript where items are reported	RECORD items	Location in manuscript where items are reported
**Title and abstract**					
	1	(a) Indicate the study's design with a commonly used term in the title or the abstract (b) Provide in the abstract an informative and balanced summary of what was done and what was found	Page 1 and 2	RECORD 1.1: The type of data used should be specified in the title or abstract. When possible, the name of the databases used should be included.<br><br>RECORD 1.2: If applicable, the geographic region and timeframe within which the study took place should be reported in the title or abstract.<br><br>RECORD 1.3: If linkage between databases was conducted for the study, this should be clearly stated in the title or abstract.	Page 1 & 2<br><br>Page 1 & 2<br><br>Page 1 & 2
**Introduction**					
Background rationale	2	Explain the scientific background and rationale for the investigation being reported	Page 3		
Objectives	3	State specific objectives, including any prespecified hypotheses	Page 3		
**Methods**					
Study Design	4	Present key elements of study design early in the paper	page 1& 2		
Setting	5	Describe the setting, locations, and relevant dates, including periods of recruitment, exposure, follow-up, and data collection	Page 3		
Participants	6	*(a) Cohort study* - Give the eligibility criteria, and the	Page 4	RECORD 6.1: The methods of study population selection (such as codes or	Page 4 & page 18

For peer review only

		sources and methods of selection of participants. Describe methods of follow-up<br>*Case-control study* - Give the eligibility criteria, and the sources and methods of case ascertainment and control selection. Give the rationale for the choice of cases and controls<br>*Cross-sectional study* - Give the eligibility criteria, and the sources and methods of selection of participants<br><br>*(b) Cohort study* - For matched studies, give matching criteria and number of exposed and unexposed<br>*Case-control study* - For matched studies, give matching criteria and the number of controls per case	Page 4	algorithms used to identify subjects) should be listed in detail. If this is not possible, an explanation should be provided.<br><br>RECORD 6.2: Any validation studies of the codes or algorithms used to select the population should be referenced. If validation was conducted for this study and not published elsewhere, detailed methods and results should be provided.<br><br>RECORD 6.3: If the study involved linkage of databases, consider use of a flow diagram or other graphical display to demonstrate the data linkage process, including the number of individuals with linked data at each stage.	Diagram 1
Variables	7	Clearly define all outcomes, exposures, predictors, potential confounders, and effect modifiers. Give diagnostic criteria, if applicable.	Page 4 & 5 & 6	RECORD 7.1: A complete list of codes and algorithms used to classify exposures, outcomes, confounders, and effect modifiers should be provided. If these cannot be reported, an explanation should be provided.	Page 4 &5 & page 12
Data sources/ measurement	8	For each variable of interest, give sources of data and details of methods of assessment (measurement).<br>Describe comparability of assessment methods if there is more than one group	Page 4 & 5 & 6		
Bias	9	Describe any efforts to address potential sources of bias	Page 5 & 6		
Study size	10	Explain how the study size was	Page 6 ; Diagram 1		

For peer review only - http://bmjopen.bmj.com/site/about/guidelines.xhtml

		arrived at			
Quantitative variables	11	Explain how quantitative variables were handled in the analyses. If applicable, describe which groupings were chosen, and why	Page 5 & 6		
Statistical methods	12	(a) Describe all statistical methods, including those used to control for confounding<br>(b) Describe any methods used to examine subgroups and interactions<br>(c) Explain how missing data were addressed<br>(d) *Cohort study* - If applicable, explain how loss to follow-up was addressed<br>*Case-control study* - If applicable, explain how matching of cases and controls was addressed<br>*Cross-sectional study* - If applicable, describe analytical methods taking account of sampling strategy<br>(e) Describe any sensitivity analyses	Page 5 & 6		
Data access and cleaning methods		..		RECORD 12.1: Authors should describe the extent to which the investigators had access to the database population used to create the study population.<br><br>RECORD 12.2: Authors should provide information on the data cleaning methods used in the study.	Page 3 &4
Linkage		..		RECORD 12.3: State whether the study included person-level, institutional-	Page 4

For peer review only

				level, or other data linkage across two or more databases. The methods of linkage and methods of linkage quality evaluation should be provided.	
**Results**					
Participants	13	(a) Report the numbers of individuals at each stage of the study (*e.g.*, numbers potentially eligible, examined for eligibility, confirmed eligible, included in the study, completing follow-up, and analysed)<br>(b) Give reasons for non-participation at each stage.<br>(c) Consider use of a flow diagram	Page 6 & 7<br>Diagram 1	RECORD 13.1: Describe in detail the selection of the persons included in the study (*i.e.,* study population selection) including filtering based on data quality, data availability and linkage. The selection of included persons can be described in the text and/or by means of the study flow diagram.	Page 6 & 7<br>Diagram 1
Descriptive data	14	(a) Give characteristics of study participants (*e.g.*, demographic, clinical, social) and information on exposures and potential confounders<br>(b) Indicate the number of participants with missing data for each variable of interest<br>(c) *Cohort study* - summarise follow-up time (*e.g.*, average and total amount)	Page 6, 7 & 8<br><br>Table 1		
Outcome data	15	*Cohort study* - Report numbers of outcome events or summary measures over time<br>*Case-control study* - Report numbers in each exposure category, or summary measures of exposure<br>*Cross-sectional study* - Report numbers of outcome events or summary measures	Page 8-15		
Main results	16	(a) Give unadjusted estimates	Page 8- 15		

For peer review only

		and, if applicable, confounder-adjusted estimates and their precision (e.g., 95% confidence interval). Make clear which confounders were adjusted for and why they were included<br>(b) Report category boundaries when continuous variables were categorized<br>(c) If relevant, consider translating estimates of relative risk into absolute risk for a meaningful time period			
Other analyses	17	Report other analyses done—e.g., analyses of subgroups and interactions, and sensitivity analyses	Page 11		
**Discussion**					
Key results	18	Summarise key results with reference to study objectives	Page 16		
Limitations	19	Discuss limitations of the study, taking into account sources of potential bias or imprecision. Discuss both direction and magnitude of any potential bias	Page 16, 17	RECORD 19.1: Discuss the implications of using data that were not created or collected to answer the specific research question(s). Include discussion of misclassification bias, unmeasured confounding, missing data, and changing eligibility over time, as they pertain to the study being reported.	Page 16, 17
Interpretation	20	Give a cautious overall interpretation of results considering objectives, limitations, multiplicity of analyses, results from similar studies, and other relevant evidence	Page 17		
Generalisability	21	Discuss the generalisability (external validity) of the study results	page 17		

For peer review only

Other Information					
Funding	22	Give the source of funding and the role of the funders for the present study and, if applicable, for the original study on which the present article is based	Page 18		
Accessibility of protocol, raw data, and programming code		..		RECORD 22.1: Authors should provide information on how to access any supplemental information such as the study protocol, raw data, or programming code.	Page 18, 19

*Reference: Benchimol EI, Smeeth L, Guttmann A, Harron K, Moher D, Petersen I, Sørensen HT, von Elm E, Langan SM, the RECORD Working Committee. The REporting of studies Conducted using Observational Routinely-collected health Data (RECORD) Statement. *PLoS Medicine* 2015; in press.

*Checklist is protected under Creative Commons Attribution (CC BY) license.

For peer review only
